# A Review on Electrospun Nanofibers Based Advanced Applications: From Health Care to Energy Devices

**DOI:** 10.3390/polym13213746

**Published:** 2021-10-29

**Authors:** Vundrala Sumedha Reddy, Yilong Tian, Chuanqi Zhang, Zhen Ye, Kallol Roy, Amutha Chinnappan, Seeram Ramakrishna, Wei Liu, Rituparna Ghosh

**Affiliations:** 1 Centre for Nanotechnology & Sustainability, Department of Mechanical Engineering, National University of Singapore, Singapore 119260, Singapore; sumedha@u.nus.edu (V.S.R.); e0702020@u.nus.edu (Y.T.); e0816507@u.nus.edu (C.Z.); e0816501@u.nus.edu (Z.Y.); mpecam@nus.edu.sg (A.C.); 2 Key Laboratory for Information Photonic Technology of Shaanxi Province, School of Information and Electronics Engineering, Xi’an Jiaotong University, Xi’an 710049, China; 3 Centre for Advanced 2D Materials, National University of Singapore, Singapore 117546, Singapore; c2dkalr@nus.edu.sg; 4 School of Instrument Science and Engineering, Southeast University, Nanjing 211189, China

**Keywords:** nanofibers, electrospinning, applications, health care, energy devices

## Abstract

Electrospun nanofibers have been exploited in multidisciplinary fields with numerous applications for decades. Owing to their interconnected ultrafine fibrous structure, high surface-to-volume ratio, tortuosity, permeability, and miniaturization ability along with the benefits of their lightweight, porous nanofibrous structure, they have been extensively utilized in various research fields for decades. Electrospun nanofiber technologies have paved unprecedented advancements with new innovations and discoveries in several fields of application including energy devices and biomedical and environmental appliances. This review article focused on providing a comprehensive overview related to the recent advancements in health care and energy devices while emphasizing on the importance and uniqueness of utilizing nanofibers. A brief description regarding the effect of electrospinning techniques, setup modifications, and parameters optimization on the nanofiber morphology was also provided. The article is concluded with a short discussion on current research challenges and future perspectives.

## 1. Introduction

**Figure 1:**
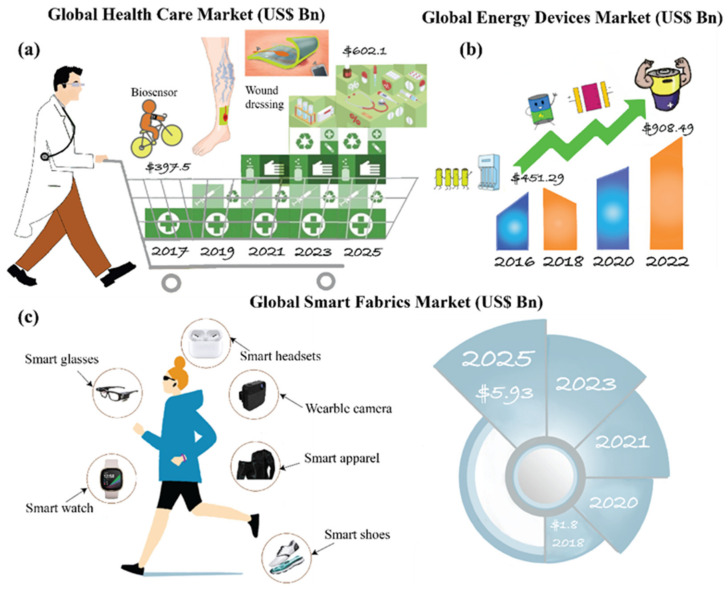
Statistics of (**a**) the global health care market (**b**) the global energy devices market and (**c**) the global smart fabrics market.

The fourth industrial revolution has remodeled various technological domains including nanotechnology, biotechnology, new materials, artificial intelligence, and human-machine interfaces. This resulted in the conjugation of nanotechnology with medicine and has also driven a new wave into the energy sector [1,2,3]. The effect of this emergence has been evidently noticed in the past two years during the period of COVID-19, when there was a smooth overall outdoor-to-indoor transition through advanced portable miniaturized technologies. Advancement in these different aspects has resulted in the overall amplification of global market value, picturized in Figure 1. In health care [4], the global market value is anticipated to increase from 397.5 to 602.1 billion USD in the timeline from 2017 to 2025 (Figure 1a), where the global energy devices market [5] has projected a 451.29 to 908.49 billion USD enhancement from 2016 to 2022 (Figure 1b) and the global smart fabrics market [6] is estimated to increase up to 5.93 from 1.8 billion USD from 2018 to 2025 (Figure 1c).

The ability to make large differences with micro/nano scale materials has been realized through nanotechnology [7,8]. The materials in the nanoscale range are a rapidly developing domain that has made a huge contribution towards revolutionizing these technologies [9,10,11]. Even though there are various nanostructured materials like nanoparticles, nanodots, nanosheets, nanorods, nanoflowers, and etc., nanofibers [12], with their unique fabrication methods and tunable properties, have become a much-explored area for various applications in the fields of sensors [13], tissue engineering [14], drug delivery [15], wound healing [16], energy devices [17], filtration [18], distillation [19], the environment, etc.

Owing to their novel physical and chemical properties in providing high specific surface area, large surface-to-volume ratio, tunable porosity, and the ability of desired chemical functionalization, they are competent enough to overcome many limitations faced in their corresponding macroscales [20]. There are several fabrication techniques such as thermal-induced phase separation [21], self-assembly [22], template synthesis [23], and electrospinning for synthesizing nanofibers. However, among these, the electrospinning technique is the most favorable choice due to the formulation into 2D and 3D structures as well as its simple, continuous, straightforward, rapid, and cost-effective process of manufacturing nanofibers [24]. Electrospinning is the most efficient way to prepare composite fibers with synergistic characteristics for new applications through blending multiple polymers with individual functionalities in the solution phase [25].

**Figure 2:**
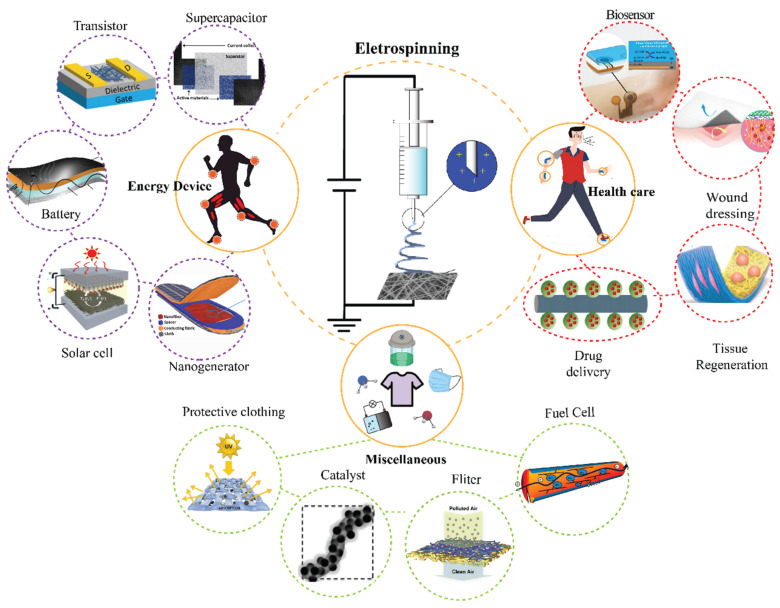
Various applications of electrospun nanofibrous materials. Health care applications [26]: biosensor [27], tissue engineering [28], wound dressing [29], drug delivery [30]; energy devices [32]: battery [33], supercapacitor [34], solar cell [35], transistor [36], nanogenerator [37]; miscellaneous applications: fabric technology [39], catalysis [40], fuel cell [41], and filtration [42].

Branched-out applications of the nanofibrous material are presented in Figure 2. Major contributions in the advancement of technologies have been observed in health care applications [26], mainly in the aspects of biosensing [27], tissue engineering [28], wound dressing [29], and drug delivery [30]. In the biomedical field, it has been recognized that the human anatomy of organs and tissues like skin, bones, collagen, cartilage, etc. can be imitated or resembled by tuning nanofiber material [31]. The large surface area is extensively exploited in these studies in enhancing adhesion to cells, proteins, and drugs when compared with the bulk materials, which gives nanofibers an edge.

Shredding some light on the environmental deterioration, climate change, and depleting fossil fuels, the dire importance of developing renewable and rechargeable energy devices is understood. Here, the nanofibers have been broadly utilized in various types of energy devices [32] including batteries [33], supercapacitors [34], solar cells [35], transistors [36], nanogenerators [37], etc. The use of nanofibers in energy conversion and storage devices has significantly drawn attention because of their ability to provide higher energy harvesting efficiency, conversion efficiency, durability, and power density due to their high surface area and controllable porosity [38]. Apart from energy devices and health care applications, there are other miscellaneous applications of nanofibers such as fabric technology [39], catalysis [40], fuel cells [41], filtration [42], and etc., which are also highly studied.

Over the past few decades, the global market value of the health care sector, energy devices, and smart fabric technologies has been escalating while crossing the thousand-billion-dollar mark. Along with that, in the near future, market value with innovation is predicted to go far beyond. This review article provided a broad survey on the contribution of electrospun technology over the past 2–3 years in these application fields through discussing the most recent developments accompanied with current and future research trends. The role of nanofibers and associated device performance as well as advantages and disadvantages of each application were emphasized in detail. Here, we anticipated the future scope and direction through summarizing the drawbacks faced in the current research scenario. This article is organized as follows: in the first section, we discuss various aspects of the electrospinning process, including setup, spinning, solution, and environmental parameters. The following section of this article focuses on the most recent research, including the past 2 years of work while emphasizing the role of nanofibers, their structure, and device performance. Finally, we conclude by addressing existing challenges which should be addressed for future perspectives.

## 2. Nanofiber Morphology and Electrospinning Techniques

### 2.1. Properties

Nanofiber materials, owing to their one-dimensional nanostructured morphology, are noticed to have superior properties when compared with bulk materials. Various unique properties such as their small size, higher carrier mobility, larger specific surface area, and high surface-to-volume ratio have helped in the emergence of nanofiber technology by introducing nanofibers as an appropriate active material for various applications in the fields of electronics, biomedicine, energy storage, healthcare, fabric technology, etc. [43]. These nanofibers can provide effective mobility as 1D path carriers by lowering boundaries with adjacent crystallite grains in flexible electronics. Their size and larger specific surface area aids faster response towards external stimuli, whereas their high aspect ratio facilitates tunable mechanical and physical properties. These remarkable properties involve tolerance towards mechanical bending or stretching [44,45], enhanced electrical conductivity [46], low density, larger specific surface area [47], good optical [48], thermal [49], optoelectronic [50], and electrical properties [51], high aspect ratio [52], good transparency [53], and convenient geometry with tunable pore volume and size [54]. For the past 100 years, researchers have been synthesizing nanofibers by electrostatically stretching viscous polymer solution into fibrous form through the electrospinning technique [55]. Usually, this method is applicable to synthetic and natural polymers, along with polymer alloys and polymers that are loaded with nanoparticles, metals, ceramics, or any other active agents [56]. Polymers possess inert chemical properties, and hence producing polymer nanofibers provides a structural benefit in tunable surface modifications for desired properties in specific applications [57].

### 2.2. Electrospinning Parameters and Their Significance

Several factors affect the nanofiber morphology and uniformity in the electrospinning process. These factors involve precursor solution parameters, process setup parameters, and environmental conditions.

Concentration, molecular weight, surface tension, dielectric constant, viscosity, and conductivity of the solution are the affecting parameters of precursor solution towards nanofiber formation [58]. Studies prove that the shape of the polymer droplet ejected through the syringe transforms from spherical to the spindle, eventually forming fibers with uniform, similar and increased diameter with an increasing solution concentration [59]. Molecular weight, polymer concentration, and viscosity are correlated and have an overall impact on nanofiber formation [60,61]. In general, polymeric molecular weight is correlated with the degree of polymer chain entanglements that allow determining the formation of either fibers with beads or clean fibers [62,63]. In the electrospinning process, higher surface tension causes instability in the jet formation at the nozzle, which results in droplet formation through spraying rather than fiber formation, and hence a solution with lower surface tension is preferred [64]. The viscosity of the solution needs to be optimum for fiber generation since extreme solution viscosity results in an unstable jet release [65]. Viscosity is varied by the proportion of solute (polymer or polymer composite) and solvent. The polymer content should be ideal for the formation of perfect nanofibers [66]. The solution conductivity is largely influenced by few key parameters: polymer type, solvent, and salt content (degree of ionization). Nanofiber diameter decreases with increasing solution conductivity, whereas a reduction in the solution conductivity leads to elongated jet formation, causing non-uniformity and bead formation or even obstruction in solution flow. On the other hand, solutions with excessive conductivity result in extreme bending instability and broad variation of fiber diameter in the presence of high voltages. For these reasons, an optimum solution conductivity is required to optimize fiber morphology as well as fiber diameter [67,68]. Besides this, the nature of the solvent is another deciding factor for spinnability and nanofiber porosity. During electrospinning, before the jet reaches the collecting platform, a phase separation takes place to form solid nanofibers. Solvent volatility can highly influence this process by modulating the effective evaporation rate. It is observed that for highly volatile solvents, pore population tends to be much higher when compared with the less volatile substances, providing increased surface area and fine surface texture [69].

In the process setup parameters, applied voltage, flow rate, and solution output nozzle-to-collector distance play crucial roles in nanofiber formation. Electrostatic repulsion force is induced on the solution jet by application of external voltage [70]. This facilitates fiber outcome, ease of solvent evaporation, and a decrease in fiber diameter which is attributed to the stretching of polymer solution in association with charge repulsion within the polymer jet [71]. Based on the polymer solution properties, the critical value of the voltage varies. As the applied voltage increases over the critical limit, there is a possibility of bead formation. Flow rate controls the amount of polymer being released for fiber formation, which requires adjusting and deciding based on the solution viscosity. Generally, lower flow rate is advised as it provides a longer time for solvent evaporation [72]. Nozzle-to-collector distance provides time for the polymer solution to convert into a fiber while facilitating solvent evaporation [69].

Apart from solution and process conditions, environmental parameters such as humidity and temperature also influence the nanofiber morphology. Temperature changes the solution viscosity, which in turn affects the spinning process [63]. Whereas, humidity effects the rate at which solution evaporation occurs [73]. For obtaining nanofibers with different morphologies, optimized parameters along with modified electrospinning setup are utilized.

### 2.3. Electrospinning Setup Modifications

A conventional electrospinning setup consists of a spinning electrode, solution holder, pushing pump, spinneret, high-voltage power supply, and a grounded collector. Since the development of the electrospinning process, several modification techniques have emerged in order to provide tailored morphology to the nanofibers. Some of these techniques involve coaxial, gas-assisted, porous hollow tube, multi-jet, self-bundling, and roller electrospinning [58,74].

**Figure 3:**
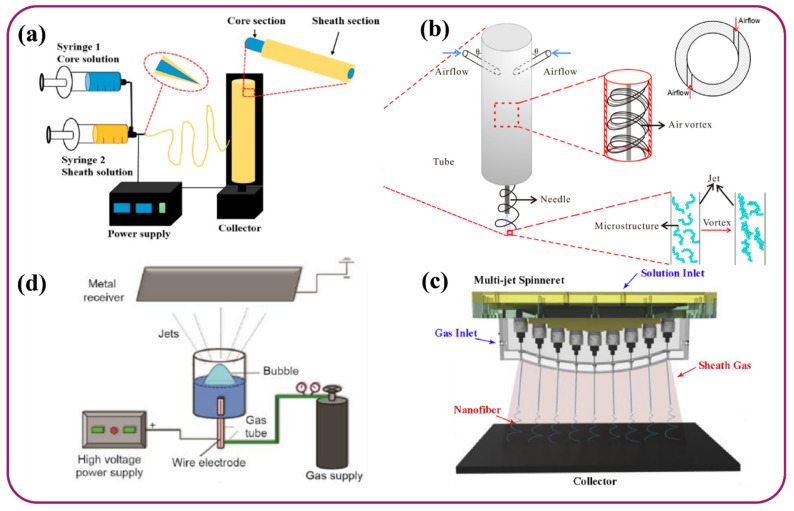
(**a**) Schematic of the coaxial electrospinning system [87]. (**b**) Illustrative set-up used in the gas-assisted electrospinning process [88]. (**c**) Experimental setup of the multi-jet electrospinning [89]. (**d**) Graphic representation of the bubble electrospinning technique [90].

Coaxial electrospinning is generally employed to fabricate core-shelled structured nanofibers. Here, two solutions are used, in which one acts as a sheath (shell) and the other acts as a core. Two syringes are used in the working with different solutions which are pushed simultaneously to release the material through their respective capillaries and facilitate formation of composite nanofibers, illustrated in Figure 3a [75]. Solution compatibility (either immiscible or semi-miscible), viscosity ratio of core-to-shell solution, and concentration are the key controlling factors for coaxial electrospinning [7]. Gas-assisted spinning is another technique that especially aids in the spinning of polymer solution with lower conductivity, which is difficult to spin in the normal setup. Figure 3b exhibits the electrospinning setup. In this technique, either high-velocity hot air or compressed air is used to make the fibers finer [76], with high surface area, barrier properties, and insulation value [77]. In multi-jet electrospinning shown in Figure 3c, the basic notion involves obtaining higher productivity by increasing the number of solution jets by implementing different methods [78]. Some of these methods involve the application of an external electric field [79], using a curved collector [80], splitting the jets to form separate sub-jets [81], and using multiple needles and needleless systems [82]. This technique enables improved production efficiency along with cost-effectiveness as a result of electrospinning through multiple nozzles [69]. Bubble electrospinning is performed by a porous hollow tube, which is a needleless process, and the polymer solution is pushed through the pores of a walled cylindrical tube that facilitates the formation of hollow nanofibers [83]. There are various ways of forming hollow nanofibers by bubble electrospinning or blown bubble spinning, in which several bubbles are generated on the solution surface using a gas pump Figure 3d [84,85]. This is one of the most promising needleless electrospinning with advantages like high productivity and lower energy consumption due to the utilization of a third force that helps in overcoming the surface tension [86].

Besides these, a few other application-specific electrospinning setups include the charge-injection method [91], near-field electrospinning [92], solvent-free electrospinning including supercritical CO2-assisted electrospinning [93], magnetic field-assisted [94], anion-curing electrospinning, and UV-curing electrospinning [95], etc.

## 3. Applications of Nanofibers

The evolution of electrospinning technology and the ability to fabricate tunable nanofibers have facilitated the development of advanced applications in the fields of health care, energy devices, the textile industry, environmental appliances, etc.

### 3.1. Health Care

Being a dominant and necessary sector, health care, or biomedical applications, is of the utmost importance and has become a highly explored area by researchers. As a potential solution to various challenges faced in the field of biomedicine, nanofiber technology is beneficial and has attractive properties. Resolutions such as organ repair, crucial vital monitoring, burns and wound dressing, blood purification, treatment for various diseases, and etc. have been offered by nanofiber technology [96].

#### 3.1.1. Biosensors

Biosensors are analytical tools that assist in the detection, quantification, and monitoring of various analytes such as enzymes, bacteria, cells, nucleic acids, antibodies, etc. present in human body [97]. Such devices require high selectivity along with sensitivity towards analytes where incorporation of nanofiber technology has demonstrated tremendous potential [98]. Using nanofibers, miniaturization of the sensing platform was made possible, which can act in both micro/nano as well as the macro-scale while providing accurate results [99]. Sensing platforms are classified based on different types of transducers such as electrochemical, magnetic, thermometric, those involving optical fluorescence, luminescence, and absorbance, electromechanical, amperometric, potentiometric, etc. [96]. In general, these devices’ performances are quantified in terms of selectivity, linearity, sensitivity, response time, reproducibility, linear range, and limit of detection [100].

In this field, researchers have utilized dimension, porosity, and the surface-to-volume ratio of nanofibers to develop highly efficient antibody detectors [101].

##### Nefastin Antibody Detection

For example, in their recent work, Kim et al. [102] developed FET (field-effect transistor) biosensors for nesfatin-1 antibody detection using multiscale pore-contained carbon nanofibers. 1D carbon nanomaterials are preferred as channel materials in FET biosensors as they can be easily tuned to immobilize the bioreceptors through π-stacking or covalent bonds. In this work, carbon nanofibers acted as signal transducers and templates for the immobilization of the nesfatin-1 antibody, achieving a low limit of detection (LOD) of 0.1 fM and a fast detection time of less than a second due to their multiscale porous structure. Electrospun fibers have also been explored for DNA detection. In a DNA molecule, guanine is the most oxidizable chemical base which is highly exploited in DNA sensing. Recently, Civan et al. [103] synthesized a hybrid cellulose nanofiber to monitor guanine-base oxidation in single-strand DNA by electrochemical methods. Because of its biocompatibility as well as its mechanical, physical, and chemical properties, cellulose is used for a variety of applications. When converted into fibrous form high surface-to-volume ratio, it provides a larger number of DNA molecules to be absorbed on the surface, supporting higher sensitivity. Here, hybrid nanofibers were synthesized by the electrospinning of cellulose monoacetate and tetraethyl orthosilicate. The fiber diameter, here, was controlled by the catalyzer and hydrochloric acid and attained between 42–958 nm. Here, the nanofiber played a role in monitoring guanine-base oxidation in single-strand DNA by electrochemical methods.

##### Glucose Sensing

**Figure 4:**
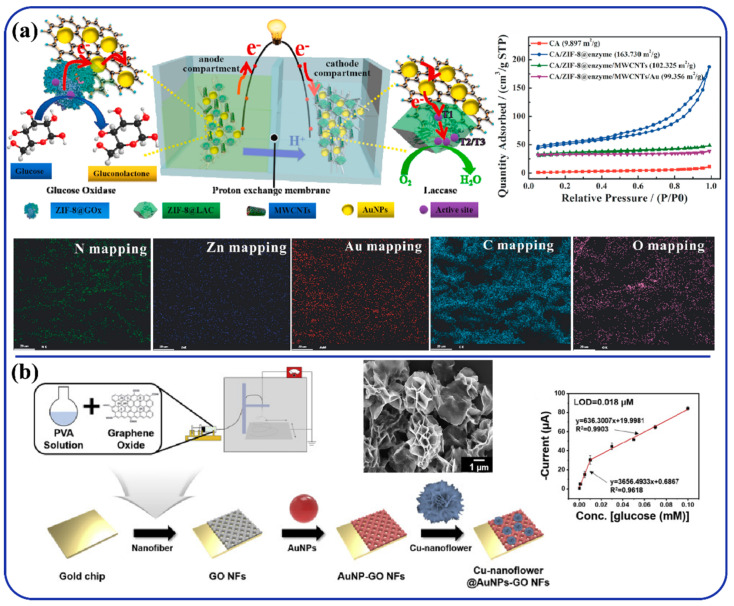
(**a**) Schematic of the self-powered glucose biosensor; SEM-EDS elemental mapping images of CA/ZIF-8@enzyme/MWCNTs/Au electrode; BET analysis of as-prepared samples [105]. (**b**) Schematic illustration for the fabrication of Cu-nanoflower/AuNPs-GO NFs-based electrochemical glucose biosensor and FE-SEM image formation of Cu-nanoflowers [107].

In the field of glucose sensing, electrospun nanofiber-based biosensors are highly explored because of their high surface area, which provides a large immobilization site resulting in increased interaction with analytes, shorter detection time, and increased lifetime [104]. A novel self-powered glucose biosensor was developed by Li et al. [105], as depicted in Figure 4a. The synthesis process involves encapsulation of the enzyme into a zeolitic imidazolate metal organic framework (ZIF-8) during in-situ growth on cellulose acetate (CA) nanofibers. In final device, highly flexible electrodes were prepared by step-by-step adsorption of MWCNTs and AuNPs on CA/ZIF-8@enzyme membranes. Here, due to its hydrophilic nature, cellulose can act as an inherent electrolyte reservoir where porous fibrous structure helps in transferring the diffused ions within electrolytes and electrochemically active materials. On the other hand, in-situ growth of ZIF-8 not only provides a successful enzyme encapsulation strategy, but also improves the enzyme stability aided by structural constraints. The addition of CNTs promotes direct electron transfer within the device. BET surface area values for CA nanofibers and CA/ZIF-8@enzyme/MWCNTs/Au were 9.879 m^2^ g^−1^ and 99.356 m^2^ g^−1^, respectively. Here, inclusion of ZIF-8 crystal within CA fiber network contributed in achieving higher porous structure and high absorption capacity. During cyclic voltammetry measurements, significant redox peaks were observed at ~0.39 V and ~0.51 V. These nanofiber membranes showed long-term stability up to 15 h. Recently, another glucose sensor was reported by Sapountzi et al. [106]. Here, polyacrylonitrile (PAN) nanofiber mats of 650 ± 10 nm diameter coated with conductive polypyrrole (PPy) layers were produced by a two-step process involving electrospinning and vapor-phase polymerization. The electrospun PAN NFs acted as a backbone with a non-conductive structure (core fibers) whilst facilitating the growth of PPy-based coatings onto their surfaces. Here, the carboxyl group nanofibers introduced into the PPy coatings enabled covalent binding of a model enzyme, glucose oxidase, which was utilized for the detection of glucose content. The device could act within a broad detection range of 20 nM–2 μM glucose concentration with a very low detection limit of 2 nM. Another electrochemical biosensor for glucose detection was synthesized by decorating Cu-nanoflower onto the gold nanoparticles (AuNPs)/graphene oxide (GO) PVA nanofiber (Figure 4b). GO acted as a fiber precursor, improving electrochemical properties of the fiber matrix. It was further decorated with AuNPs via electrostatic interaction to increase electrical conductivity and detection sensitivity. Finally, the surface coating of unique organic-inorganic nanostructured materials (Cu-nanoflower) enhanced catalytic properties. These composite nanofibers facilitated in yielding an efficient electro-catalyzed reaction that converted glucose to gluconic acid. The final device, Cu-nanoflower@AuNPs-GO NFs-coated Au chip, exhibited a wider linear range (0.001–0.1 mM) with LOD of 0.018 μM [107].

##### β-Amyloid Detection

Another type of electrochemical sensor has been recently developed by Supraja et al. [108] using electrospun Tin oxide (SnO_2_) nanofiber. These were used for label-free detection of β-Amyloid (1–42) in early-stage diagnosis of Alzheimer’s Disease. This hormone-specific capture antibody was covalently immobilized on the carbon electrode modified by SnO_2_ nanofibers. SnO_2_ is an intrinsic n-type semiconductor containing a number of oxygen vacancies present at the grain boundaries. These vacancies enable absorption of external molecules which increases Schottky barrier height, resulting in a reduction of conductivity which in turn controls the electrical behavior of the sensor. This inherent heterogeneity of SnO_2_ can facilitate target analyte detection in fairly low concentrations of analyte. It has been noted that within the concentration range of 1 fg/mL^−1^ μg/mL, the sensing platform shows a sensitivity of 274.96 (kΩ/ng.mL^−1^)/cm^2^ and 0.146 fg/mL LOD.

##### Levodopa Detection

In another study Wang et al. [109] used Molybdenum disulfide (MoS_2_) nanosheet arrays/carbon nanofibers. These nanofibers were used as an electrode in a biosensor for detection of levodopa and uric acid. MoS_2_ nanosheet arrays are promising materials for sensing applications due to their large surface area, excellent conductivity, high catalytic efficiency, and biocompatibility. When combined with nanofibers, the composite material possessed high electronic conductivity with improved electrochemical active sites by increasing the specific surface area of CNFs network. These composite nanofibers facilitated in achieving stability, good repeatability, and higher sensitivity (0.91 μA μM^−1^ for levodopa and 0.73 μA μM^−1^ for uric acid) within the range of 1–60 μM.

##### H_2_O_2_ Detection

In the field of biosensing, hydrogen peroxide (H_2_O_2_) detection plays an important role for disease determination, as it is a common by-product of some biochemical processes, catalyzed by enzymes (such as glucose oxidase, cholesterol oxidase etc.) and triggering cancer growth. Daemi et al. [110] worked in a non-enzymatic biosensor for H_2_O_2_ detection with ZnO-CuO hybrid nanofiber that has a sensitive amperometric response and LOD of 2.4 µM. Though ZnO is a low-cost inorganic n-type semiconductor with a wide band gap of 3.37 eV, high mobility, and non-toxicity, it suffers from weak cycling performance when used as an electrode material. To address this issue, CuO (a p-type stable semiconductor with narrow band gap 1.2–1.9 eV) was incorporated to synthesize a hybrid nanostructure which provided synergetic electrochemical sensing properties due to the presence of binary metal oxides. This hybrid nanofiber exhibited the lowest charge transfer resistance and the highest electrocatalytic performance among other modified electrodes used for the detection of H_2_O_2_.

Among various biosensing platforms, FETs have diverse applications in the detection of enzyme, immune, DNA, cell-based materials with advantages like fast response speed, accurate detection, low cost, and low power consumption. Recently, Hong et al. [111] fabricated In-Ga-Zn-O nanofiber-based double-gate FETs biosensors for chemical sensing with high sensitivity (998 and 383 mV/pH, pH sensitivities for In-Ga-Zn-O nanofiber and In-Ga-Zn-O film-based sensors respectively). Here, In-Ga-Zn-O nanofiber was synthesized by electrospinning of In-Ga-Zn-O precursor along with polyvinylpyrrolidone (PVP) polymer. Unlike the conventional chemical vapor deposition or solution-processing methods, here, electrospinning was employed to synthesize In-Ga-Zn-O nanofibers that improved a capacitive coupling effect. These fibers were applied to the channel layers to increase the sensitivity without adjusting the gate-oxide materials or thickness.

Depending on the origin of the polymers, these biosensors can also be classified into two major categories: natural and synthetic polymer-based biosensors, as shown in Table 1. These studies include natural polymers like cellulose, CA, etc., and synthetic polymers like PVA, PAN, PPY, PVP, etc. for developing biosensors for their biocompatibility, biodegradability, and non-toxic nature.

#### 3.1.2. Tissue Regeneration

Regenerating the growth of cells and tissues is a part of tissue engineering that aims to repair or replace function to the damaged tissues through biological principles [69]. The major challenge in tissue engineering is the development of scaffolds that can replicate tissue architecture at the nanoscale level. Nanofibers have been used as basic building blocks for tissue-regenerating scaffolds for the past few decades. They serve as an excellent framework for cell adhesion, proliferation, and differentiation by meeting existing challenges [114,115].

Among various existing techniques, electrospun nanofibers are most widely used for tissue engineering applications due to their higher surface area and porosity, which aid as favorable factors for cell interactions [116,117]. Both natural and synthetic materials possessing biocompatibility, biomimicking, hydrophilicity or hydrophobicity, polymer-drug adhesion, and control over drug release have been extensively explored for these applications [114,118]. The nanofibers possessing these qualities are utilized in various types of tissue regeneration applications such as musculoskeletal (involving bone, ligament, cartilage, and skeletal muscle), vascular, skin, neural, as carriers for the controlled delivery of drugs proteins, and DNA [119]. Few contemporary studies in the field of tissue regeneration through integration of nanofiber technology were discussed here.

##### Bone Tissue Regeneration

**Figure 5:**
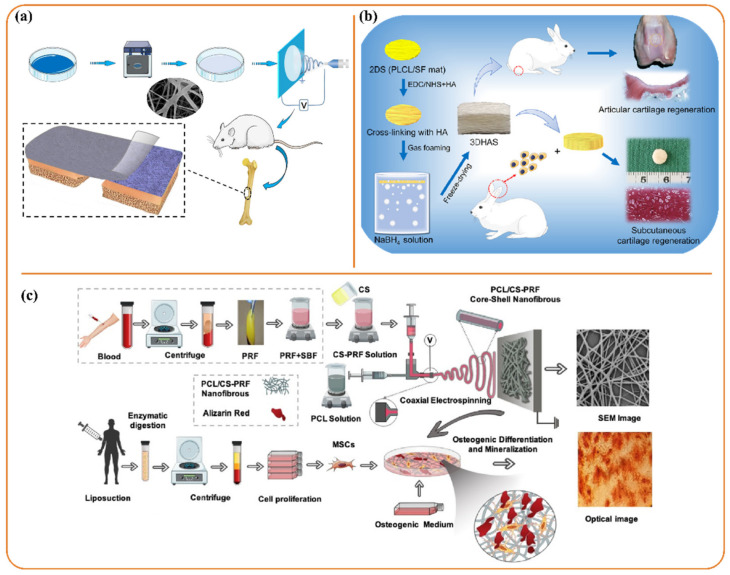
(**a**) Representation of the method of preparation and implantation of the bilayer membrane [121]. (**b**) Illustration of scaffolds synthesis their application for cartilage tissue engineering [123]. (**c**) Schematic of preparing core-shell nanofibrous scaffold and osteogenic differentiation of human mesenchymal stem cells into osteoblasts on the scaffolds [122].

Bone tissue regeneration is a complicated procedure which depends on several factors that involve maintaining the defect shape while preventing any further compression to the initial blood clot, along with the capability to shield soft tissues cells infiltration during maturation of the bone matrix. At the same time, maintaining suitable mechanical properties for required regeneration timeline withholding appropriate biodegradability is also needed to avoid post-surgical complications [120]. Recently, Zhang and team [121] produced a biodegradable synthetic scaffold for stimulating bone tissue repair. In this study, a mineralized collagen and chitosan cast film was coated with polycaprolactone (PCL)/polyvinylpyrrolidone (PVP) electrospun nanofibers and loaded with berberine (drug), which yielded a bilayer membrane (Figure 5a). PCL, being a synthetic polymer, has good elasticity as well as biodegradability and biocompatibility, which make it suitable for tissue engineering applications. However, it suffers from low water absorption capacity and hydrophobicity. Hence, by combining it with PVP, which is a water-soluble polymer, the biodegradation and hydrophilicity of PCL can be improved. The bilayer membrane of uniform diameter (548 ± 150 nm) composite nanofibers showed favorable mechanical properties (Young’s modulus 120 MPa) while enhancing the attachment and proliferation. For testing the practical applicability, this scaffold was implanted within a rat femoral bone defect for in vitro attachment, proliferation, and regeneration of MC3T3-E1 (osteoblast precursor cell line) cells.

Among various types of scaffolds, coaxial structures provide an environment conducive to bone tissue regeneration, as they can encapsulate proteins, growth factors, drugs, and other bioactive substances. As in these structures core solution does not need spinnability, these substances are incorporated within the core structure which is released in a sustained manner for controlled therapy [14]. With the help of the coaxial electrospinning method, Rastegar et al. [122] fabricated a platelet-rich fibrin loaded/chitosan (CS) core-shell nanofibrous scaffold. CS holds various outstanding properties including biocompatibility, biodegradability, and antibacterial, which are required for biomedical applications. However, it cannot be electrospun alone and thus should be blended with some polymers like PCL which has high solubility in organic solvents and slow biodegradation. The core-shell nanofibrous structure enhanced osteogenic differentiation of mesenchymal stem cells (ability to self-renew and differentiate into multiple cell) (Figure 5c) in this study. By loading fibrin to the nanofiber structure, elastic modulus and specific surface area of the scaffold were observed to increase from 25 to 44 MPa and 9.98 to 16.66 m^2^/g respectively.

##### Articular Cartilage Regeneration

Cartilage degeneration is one of the most common joint diseases. Due to the absence of blood vessels and the innervation network, the mature articular cartilage regeneration rate is poor, which may lead to various disabilities, affecting quality of life. Recently, Chen et al. [123] engineered a gas-foamed extracellular matrix mimicking a 3D porous electrospun nanofiber scaffold for articular cartilage regeneration. In this study, 2D electrospun poly(L-lactide-co-e-caprolactone) (PLCL)/silk fibroin (SF) scaffolds were fabricated by a dynamic liquid support electrospinning system which was then cross-linked with hyaluronic acid to mimic the microarchitecture of native cartilage (Figure 5b). PLCL-based scaffolds are extensively researched for tissue engineering applications due to their mechano-elastic and biodegradable properties which impart mechanical stability in the scaffolds. Further addition of SF improves the hydrophilicity and cytocompatibility of the hybrid nanostructures, meeting requirements for the optimal cartilage regeneration. For the real-time application, the scaffold was implanted within a rabbit model. It was observed that these fabricated 3D scaffolds showed satisfactory proliferation and phenotypic maintenance of chondrocytes while exhibiting good hemocompatibility, cytocompatibility, biocompatibility, and low density, high porosity with a large pore area, good water absorption capacity, and mechanical stability.

##### Periosteal Regeneration

Found around the cortical bone, periosteum is a dense layer of vascular connective tissues, osteoprogenitor cells, and stem cells. It plays an important role in repairing bone defects. However, existing artificial periosteum materials suffer with poor mechanical properties and bionic structure construction, osteogenic differentiation, and vascularization capabilities. In order to address this issue, Zhang and group [124] prepared poly-ε-caprolactone (PCL)/whitlockite through electrospinning technology. PCL is an FDA-approved synthetic polyester material with good processability and biocompatibility and moderate degradation velocity which, is highly suitable for long-term in vivo usage. However, it lacks inherent osteoinductive abilities, which limits its application. This can be improved by introducing calcium phosphate (CaPs) like whitlockite. Whitlockite is the second-most-present CaP in natural bone tissues which inhibits osteoclast activity, promoting angiogenesis. By testing this material in vitro mineralization experiments, the rapid ion release from whitlockite was observed to promote the deposition of mineralized hydroxyapatite for human umbilical vein endothelial cell angiogenesis and migration along with collagen formation and calcium deposition.

##### Dural Regeneration

Dura mater is the outermost layer of meninges that surrounds brain and spinal cord. It provides cover and support to the dural sinuses and carries blood from the brain to the heart. For healing and regeneration of this layer, a radially aligned nanoscale surface is desirable as an artificial dural substitute. Hence scaffolds constructed with electrospun nanofibers could meet such a demand by guiding and enhancing cell migration from the edge of a dural defect to the center [125]. The most commonly used synthetic dural substitutes experience local tissue rejection or inflammation reactions induced by their acidic products, which causes degradation by forming foreign-body giant cells through monocyte/macrophage fusion. Hence, to achieve efficient dural healing, high molecular weight poly(4-hydroxybutyrate) (P4HB) polymer-based nanofiber membranes were synthesized via electrospinning, which exhibited high elasticity, biocompatibility, and long-term strength retention properties. A cyto-compatible platform in which fibroblasts can migrate, adhere, and proliferate with the lower limit of 2.15 EU (endotoxin)/devices contacting cerebrospinal fluid was successfully achieved with other characteristics such as conformability, wettability, and strength to an underlying tissue surface, for dural tissue repair. In a rabbit duraplasty model in vivo, the membrane could form a continuous neodural tissue mimicking native dura mater, which produced a watertight closure to prevent cerebrospinal fluid leakage without systematic or local infection [126].

Olfactory ensheathing cells comprise a type of glial cell which can penetrate the transition zone between the peripheral and central nervous systems. They play a key role in central nervous system repair as seed cells. Many studies have reported that the diameter of electrospun nanofibers has a significant effect on cell behavior along with cell adhesion, viability, morphology, differentiated functions, and proliferation [127,128]. Castano and group [129] produced functionalized nanoscale meshes of PLA nanofibers, seeded with chemotactic TEG3 cells, an olfactory ensheathing-derived cell line to study its attachment, morphology, and directionality in neural regeneration. The less rigid and more amorphous topographies of PLA nanofibers, which are functionalized with different concentrations of a chemotactic agent, guided the migration of TEG3 cells toward more chemokine-concentrated surfaces better than the other polymer scaffolds. It was observed that the size of the nanofibers has a strong effect on TEG3 cell adhesion and migration; here, nanofibers with 950 nm diameter showed highly dynamic behavior in migratory terms. The roles of different nanofiber materials used in various tissue regeneration applications are summarized in Table 2.

#### 3.1.3. Drug Delivery System

A drug delivery system is a formulation that facilitates therapeutic material to selectively reach the site of action without disrupting the functioning of other human body systems. Drugs are usually toxic when administered at the wrong doses. Hence, for vital cases, customization is required, which cannot be provided with conventional medication such as injections, pills, syrups, etc. Drug delivery through smaller-size suitable coating material improves their capability to be digested or absorbed by the targeted site in a much feasible manner. Electrospun fibers with controllable material composition and structural properties such as fiber diameter, geometry, and their flexibility to be formulated in various shapes effect the release profile of bioactive molecules. These properties facilitate targeted and controlled drug delivery [15,118]. The nanofibers are also capable of encapsulating labile molecules for rendered drug delivery by enhancing their stability in harsh biological environments [134].

**Figure 6:**
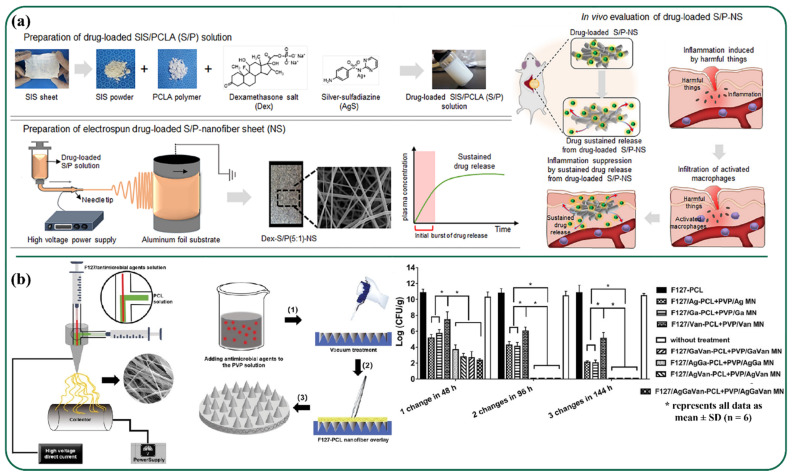
(**a**) Preparation and evaluation of drug-loaded nanofiber [135]. (**b**) Graphical representation of the fabrication of scaffolds; preparation of antimicrobial agents/core–sheath nanofiber mats using coaxial electrospinning; efficacy of single and multiple antimicrobial agents containing biphasic scaffolds against mixed-MRSA/P. *aeruginosa* biofilms using different administration strategies [137].

A few of the current innovations in the field of drug delivery were detailed in this section. Recently, Thao et al. [135] prepared an electrospun small intestinal submucosa (SIS) and poly(caprolactone-co-Lactide-co-glycolide) (PCLA) nanofiber sheet, which acted as a potential drug (dexamethasone and silver sulfadiazine) carrier related to anti-inflammation (Figure 6a). Here, amongst several synthetic polyesters, PCLA was chosen, as it offers several potential advantages like ease of preparation and better in vivo controllability due to biodegradable and biocompatible properties. The ratio between SIS and PCLA was varied from 1:1 to 5:1 to obtain various porosities (50% to 75%), pore size (ranged within 20–60 µm), fiber diameters (ranging from 380 to 100 nm), and the tensile strength (ranged within 0.75 to 0.15 N) of the fibers for optimized performance. The produced nanofiber sheet exhibited good biocompatible, bioresorbable, and non-immunogenic properties. Sustained release of the drugs while suppressing the macrophage infiltration was ensured through in vitro and in vivo studies. In another study, PVA/soy protein isolate (SPI) nanofiber mats were produced as drug carriers. PVA is a nontoxic water-soluble polymer that shows high mechanical properties and good film-forming capability. It has been reported that SPI has been widely used in many biomedical applications, as it has the capability to decrease the proliferation of tumor cells and reduce the activities of immunocompetent cells and bone-resorbing cells. Hence, for achieving synergic properties, PVA/SPI nanofibers were utilized in this study. The synthesized mats were loaded with ketoprofen by dissolving the drug in the solutions for nanofiber during electrospinning. To improve drug-release control of the nanofiber mats, a natural tubular nanoparticle named sepiolite was utilized as a secondary release-control tool. Here, three types of nanofiber mats were fabricated by direct mixing of PVA, SPI, and ketoprofen (average fiber diameter 137 nm; specific surface area 153.36 m^2^/g); direct mixing of PVA, SPI, sepiolite, and ketoprofen (average fiber diameter 117 nm; specific surface area 168.24 m^2^/g); and mixing PVA, SPI, and ketoprofen-preloaded sepiolite (average fiber diameter 129 nm; specific surface area 194.27 m^2^/g). Experiments showed that incorporation of SPI and sepiolite into the PVA nanofibers increased the mechanical strength of the mats up to 40%, making them long lasting and easier to handle [136].

A research team from the University of Nebraska fabricated biphasic scaffolds by integrating nanofiber mats with dissolvable microneedle arrays (with 100 to 150 needles on a 6 mm disk) through coaxial electrospinning for simultaneous drug delivery (Figure 6b). This type of delivery system facilitates a complementary killing mechanism exhibiting synergistic efficacy. This scaffold was reported as an antimicrobial delivery system for the effective treatment of bacterial biofilms. In this study, polyvinylpyrrolidone (PVP) microneedle arrays and Pluronic F-127-poly(ε-caprolactone) nanofiber mats were exploited with incorporation of different combinations of antimicrobial agents (AgNO, Ga(NO_3_)_3_, and vancomycin) where a biphasic scaffold served as the delivery agent for the eradication of mature biofilms on artificial wounds created on ex vivo human skin explants [137].

A different study involved the production of dual drug delivery of vancomycin and imipenem/cilastatin through core-shell nanofibers composed of polyethylene oxide (PEO), chitosan, PVP, and gelatin (with average fiber diameter varying from 218 to 342 nm) for the treatment of diabetic foot ulcer infections. For such types of wounds, methicillin-resistant *Staphylococcus aureus* is one of the main sources of infection, which can be treated using comprehensive drugs like vancomycin and imipenem. For controlled drug delivery, these drugs can be incorporated within the nanofiber matrix to reduce negative side effects and hazards like nephrotoxicity, cytotoxicity, and hypersensitivity reaction. In this work, a dual drug-loaded nanofiber was prepared using PEO, CS, and vancomycin as shell materials and PVP, gelatin, and imipenem/cilastatin as the core-forming matrix which can perform a simultaneous release of vancomycin and imipenem at different rates into the wound sites. The results showed significant antibacterial activity against methicillin-resistant *Staphylococcus aureus*, *Escherichia coli*, and *Pseudomonas aeruginosa* with no recorded cytotoxicity [138].

Bai et al. [139] synthesized ibuprofen (IBU)-541 loaded PVP nanofibers utilizing a coaxial solid-core spinneret. IBU is a widely used drug for its anti-inflammatory, analgesic, and antipyretic properties. In this study, testing of fast dissolution of IBU from its electrospun hydrophilic polymer nanocomposites was carried out. Here, the PVP nanofiber matrix was chosen as drug carrier as it has a fine physiological compatibility that does not have any stimulation to skin, mucous membrane, eyes, etc., and can act without interfering in the human metabolism. These nanofibers were observed to have an average diameter of 740 ± 130 nm with linear morphology, large surface cross-section, and porosity without any solid phase separation, providing synergistic effects with an amorphous state of the drug. Liu et al. [140] fabricated bifunctional copper sulfide (CuS)/PVP/gelatin composite green synthesized nanofibers by portable in situ electrospinning for rapid hemostasis and ablate super bacteria. CuS exhibited strong absorption in NIR irradiation, which exerts an effective bactericidal effect on super bacteria. However, it shows cytotoxicity which can be inhibited by incorporating it within a biodegradable and water-soluble polymer like gelatin and PVP, which helps in avoiding immediate contact between CuS nanoparticles and cells. This composite fiber can be used to excite surface-plasmon effects that provide greater depth of penetration into the skin. It also effectively prevents water superheating and skin damage caused by UV light. Here, nanofibers were directly deposited onto the wound to accelerate the hemostatic effect and improve the adhesion between the membrane and skin. The nanofibers with average diameter of 400 nm were produced on the wound site and showed better compactness which facilitated the shortening of the overall healing time by accelerating hemostasis to less than 6 s.

Precise drug delivery to tumor sites in cancer treatment is crucial for improved therapeutic efficacy and minimizing adverse effects, which is an unmet clinical need [141]. Gastric cancer is the third leading cause of death worldwide. Recently, to improve the chemotherapeutic performance in gastric cancer, a study was carried out by Anothra and team [142]. To treat such a cancer, 5-FU was extensively used in clinical practice. In this work, 5-FU loaded Eudragit S-100 composite nanofibers (diameter ranging from 150–180 nm, porosity of 78%, and tensile strength 170 g/cm^2^) were synthesized and optimized through a single-nozzle electrospinning technique. Eudragit S-100 was chosen because of its polyanionic and biocompatible nature which can exhibit controlled swelling in a gastric environment, providing customized drug release. It is water insoluble, as well, as an acidic and is considered as a food-grade polymer due to its non-toxicity. The developed system showed superior thermal stability, tumor regression potential, and pharmacokinetic profile when compared with the plain drug with 90% release of encapsulated drug therapeutic after 12 h, making it a potential approach for localized treatment of gastric cancer. The roles of different nanofiber materials used in various drug delivery applications are summarized in Table 3.

#### 3.1.4. Wound Dressing

Wound dressing is a process of regenerating dermal and epidermal tissues and a vital step for rapid healing of the affected part while offering protection against infection due to environmental exposure. A number of biochemical actions/phases such as proliferation, homeostasis, inflammation, and remodeling are involved in this dynamic activity [146]. For these purposes, drug-embedded nanofibers are potential substitutes in wound healing due to surface functionality, ability in inhibiting microorganism contact, oxy-permeability, and lasting drug release [118,147]. As chronic wounds are prone to infection that might cause sepsis and affect the overall healing process, studies have revealed that nanofibers can offer enhanced cell proliferation, migration, and angiogenesis for effective wound healing [148]. In this regard, recently, lots of research has been carried out on multifunctional wound dressing, which can provide all the requirements at a time for effective wound healing in order to boost restoration within the wound site and prevent undesirable effects such as infection. In these kinds of studies, nanofiber scaffolds are produced by blending various natural or synthetic polymers and incorporating drugs, nanoparticles, and bioactive agents (as growth factors, vitamins, and anti-inflammatory molecules) through the electrospinning process [149]. A few of the recent works published on the benefits of using electrospun nanofibers in wound dressing were summarized here.

*Artemisia annua* L. is a type of annual herb that has been used for various treatments and wound healing [150]. In their recent study, Peng and team [130] utilized Artemisinin (ART), a sesquiterpene lactone compound with therapeutic properties like anti-inflammatory and antibacterial activity, to evaluate in vivo wound healing in a rat model with dorsal full-thickness wound defects. Polylactic acid glycolic acid (PLGA)/silk fibroin (SF) membranes loaded with ART composite nanofibrous membranes were electrospun for this study. PLGA NFMs is a biodegradable polymer with good mechanical properties along with controlled degradability and drug release quality, which are necessary in biomedical applications. However, there are some drawbacks like poor hydrophilicity and cell affinity which can be improved by combining it with natural polymers like SF. SF, a *Bombyx mori* cocoon extract, has good cytocompatibility and biocompatibility in both in vivo or in vitro applications. SF possesses attractive characteristics such as cell adhesion and proliferation that promotes wound healing. The fabricated membrane showed breaking strength ranging from 4.0 ± 1.0 MPa to 7.4 ± 0.3 MPa and Young’s modulus from 215.3 ± 2.0 MPa to 394.9 ± 1.3 MPa with elongation at break from 34.0 ± 7.8% to 127.0 ± 7.0%. The study proved that the obtained mechanical and anti-inflammatory property was suitable for wound dressing, without cytotoxicity. This provided a new method for the development and application of ART for wound healing.

**Figure 7:**
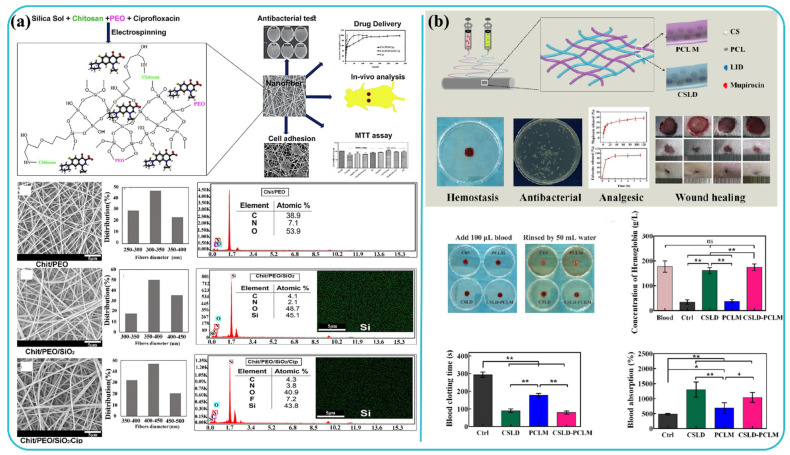
(**a**) Interactions of the inorganic framework with chitosan and polyethylene oxide; SEM images, fibers size distribution, EDX and X-ray mapping [152]. (**b**) Schematic Illustration of fabrication process of nanofiber scaffolds; concentration of hemoglobin in blood clots on scaffolds; blood clotting time and blood absorption capacity of the medical gauze control and PCLM, CSLD, and CSLD-PCLM nanofiber scaffolds * *p* < 0.05, ** *p* < 0.01 [155].

Chronic wounds, such as diabetic ulcers and burns, are complex wounds with slow healing rates, and when left untreated for longer periods of time become fatal and may result in hospitalization. In such cases, *Blumea balsamifera* (BB), which is also known as “Sambong”, is one of the essential oils which have been used for thousands of years in Southeast Asian countries. Ullah et al. [151] prepared ultrafine, bead-free, bioactive sambong oil-loaded electrospun cellulose acetate nanofibers for wound dressing applications. The as-synthesized fibrous materials exhibited in-vitro biocompatibility, cell viability, breathability (moisture vapor transport rate of 2450–1750 g/m^2^/day), and good antibacterial and antioxidant properties. The inclusion of BB oil resulted in a decrease in tensile strength and increase in fiber diameter, reducing porosity of the materials. They were also observed to undergo first-order release kinetics immediately after biphasic release. Ciprofloxacin is another well-known antibacterial drug that has been utilized for healing a wide range of wound infections. A study involving in vitro and in vivo evaluations of ciprofloxacin (antibacterial drug)-loaded chitosan/polyethylene oxide/silica nanofibers fabrication through electrospinning for treating cutaneous wounds of Balb/C mice was carried out by Hashemikia and coworkers (Figure 7a). It was noticed that fibers’ structural integrity was intact for more than 7 days in the phosphate buffer saline, while demonstrating excellent antibacterial properties. The synthesized nanofiber scaffold was able to absorb water while maintaining morphological integrity during drug release and degradation processes. It was observed here that silica played a crucial role in collagen creation, accelerating the wound-healing process through degradation products of silica-materials [152].

Annatto is a natural dye obtained from tropical plants and is mainly studied for its anti-inflammatory, antioxidant, antimicrobial, and anti-cancer activity and potential applications in the fields of wound healing and tissue engineering. A study was conducted by Santos et al. to explore wound healing efficacy, fibroblast cellular proliferation, and in vitro cytotoxicity by evaluating annatto functionalized cellulose acetate nanofiber scaffolds in a rat model. Here, the bioactive molecule included in the nanofiber and fibroblast cells was attached and penetrated to modulate the in vivo inflammatory process favorably. In the analysis, cytotoxicity found no irritation produced in hen’s egg-chorioallantois membrane test assays, which verified biocompatibility. It was observed to be viable in mouse fibroblasts even after 48 h of culture [153]. Mupirocin is an antibacterial agent that effectively reacts with the pathogens and treats various topical wounds like burns and foot ulcers [154]. Yang et al. [155] worked on designing multifunctional lidocaine hydrochloride (LID) and mupirocin-loaded chitosan/polycaprolactone (CSLD-PCLM) scaffolds using dual spinneret electrospinning and studied its varied dual drug release capability (Figure 7b). Nanofibrous scaffolds showed rapid release of LID, sustained release of mupirocin, and enhanced interfacial interaction with blood cells while possessing blood coagulation capacity. Re-epithelialization along with collagen deposition in the skin defect model of rat was also reported.

Melatonin is a hormone that exhibits positive effects on wound healing. It possesses abilities such as stimulation of fibroblasts, epithelial cell proliferation, and growth factors for collagen fibers formation [156]. A three-layer chitosan-polycaprolactone/polyvinylalcohol melatonin/chitosan-polycaprolactone wound dressing loaded with melatonin was fabricated by Minmajidi and group [157] to obtain 45% reduced burst release and sustained melatonin release for 11 days. The scaffold was tested on a full-thickness excisional model of rat skin by the local administration of the drug. During a similar timeline, Saatchi et al. [158] studied five different electrospun chitosan-based scaffolds with a wide range of cerium-doped bioactive glass-loaded chitosan/polyethylene oxide nanofiber ratio for wound-healing applications. Increasing the content of cerium doped bioactive glass enhanced the swelling degree and mechanical properties (break elongation 28.6%) of the scaffolds. The roles of different nanofiber materials used in various wound dressing applications are summarized in Table 4.

### 3.2. Energy Devices

The increase in energy demand for sustained economic growth with the simultaneous depletion of fossil fuels and emerging renewable energy is growing rapidly. Nowadays, innovation and sustenance of such renewable energy technologies/devices has become of dire importance. Some of the renewable energy sources comprise solar, wind, tidal, geothermal, etc. In energy management and usage, various issues like maximization, prolonged dependence, stability, etc. have paved the scope for energy storage technologies like fuel cells, solar cells, batteries, nanogenerators, and supercapacitors, etc. In this field of research, nanofibers synthesized through electrospinning techniques have come provide unique properties such as high surface areas and porosities, which are widely exploited for energy conversion, harvesting, and storage. Recent studies on nanofiber-based energy storage devices are discussed in this section [17,103,104,105].

#### 3.2.1. Batteries and Supercapacitors

Batteries are power sources that are either rechargeable or single usage. Some of their essential requirements include a porous, sponge-type structure of electrodes and separators to enable high discharge current capacity and allow the free exchange of ions (high ion conductivity and permeability) while efficiently preventing short circuits, respectively. Along with fulfilling the above requisites, nanofibers can also provide mechanical strength and high electrochemical stability with improved cycle life through well interconnected porous membranes [163,164]. Some of the current research works in battery applications are reported below.

In battery technologies, Lithium-ion batteries (LIBs) are the most researched area due to their high volumetric, gravimetric energy density with light weight and good shape versatility, which has led to various miniaturized applications in electrotonic as well as electrical appliances. However, achieving high power capability along with high energy density along the way remains a challenge. Here, electrospun nanofibers are implemented to overcome these difficulties by enhancing electric and ionic conductivities of LIBs through utilizing high specific surface area, porosity, controllable fiber diameter, and well interconnected porous structure. Moreover, they can be functionalized to have controlling ability over properties like electrolyte affinity, pore size, and thermal stability to achieve enhanced cell performance [165].

**Figure 8:**
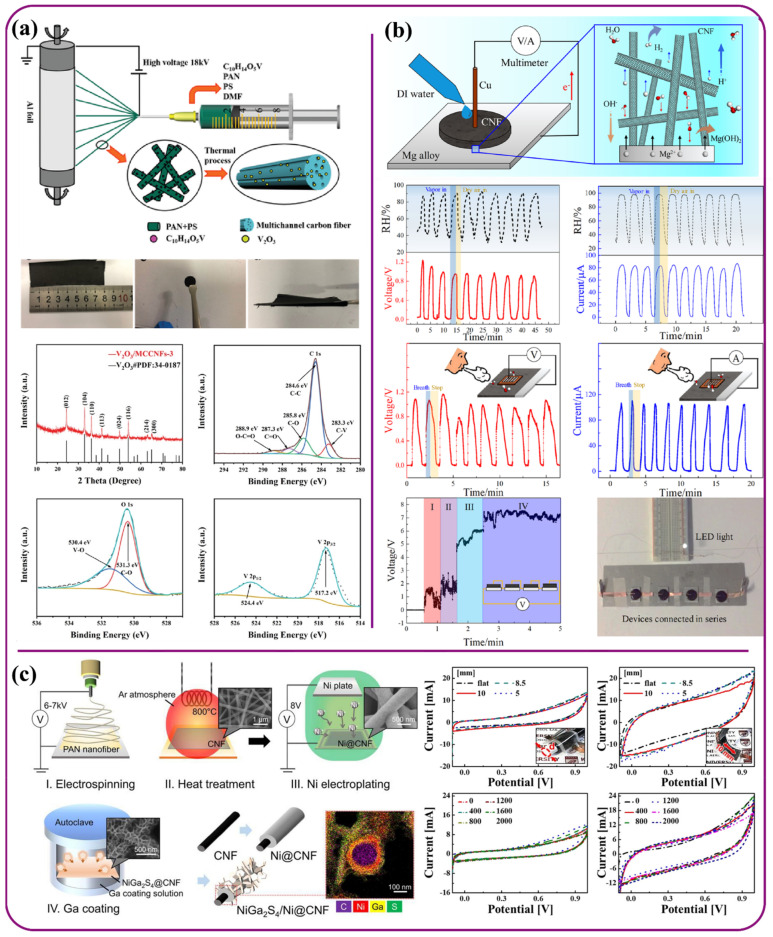
(**a**) Schematics on formation process of V_2_O_3_/MCCNFs composites; free-standing films prepared by electrospinning; displaying flexibility and 180° folding films [167]. (**b**) Illustration of the device used for water detection, as well as the Mg–water reactions and ion transportation happening in the CNF porous structure; pulse signals in response to relative humidity changes by the human breath detected by the device [169]. (**c**) Schematic of the stepwise fabrication process of NiGa_2_S_4_/Ni@CNF; electrochemical characteristics of flexible supercapacitors, CV curves with varying curvature radii (mm) with liquid and solid electrolyte [173].

Electrospun aramid fibers have been utilized in batteries as solid-state electrolyte materials to achieve high ionic conductivity along with mechanical stability, which are the prerequisites for solid-state LIBs. In their latest work, Liu et al. [166] used aramid (ANFs) nanofibers/polyethylene oxide (PEO) Lithium bis(trifluoromethanesulfonyl)imide (TFSI) as solid-state electrolyte to fabricate LIB. The composite material consisting of ANFs and polyparaphenylene terephthalamide (PPTA) molecules possess characteristics like large aspect ratio, high mechanical strength thermo-decomposition temperature, abundant amide groups, light weight, low electrical conductivity, and high chemical inertness, which enhances material strength. On the other hand, soft PEO polymer was used as electrolyte material to dissolve Li salts. Here ANFs were utilized as nano additive organic fillers in the composite electrolyte design achieved through hydrogen-bond interactions between ANFs, PEO chains, and TFSI anions. These, in turn, facilitated prolonging ion transport paths and provided a superior conductivity of 8.8 × 10^−5^ S cm^−1^ and cycling stability of 135 mAh g^−1^ after 100 cycles at 0.4 C. Comprehensive upgradation of the electrolyte was observed through obtaining higher ion conductivity, mechanical and electrochemical stabilities, and interfacial resistance against Li dendrites. For portable, wearable electronics, development of flexible and foldable LIBs that provide shorter charge and longer discharge time have received increasing attention. Traditional LIBs usually lack in the area of flexibility due to their weight and rigid built-up characteristics. Hence, to address such an issue, a study involving free-standing and foldable vanadium oxide/multichannel carbon nanofibers (V_2_O_3_/MCCNFs) composites prepared via electrospinning (Figure 8a) was carried out. Usually, in the traditional slurry-casting electrode preparation at high mass loading, materials are easily peeled off from the current collector after several enfoldments, which results in rapid capacity decay. This issue is resolved through using flexible electrodes, which possess low cost, foldability, and lightweight features. In this study, a flexible structure formed by loading V_2_O_3_ particles on MCCNFs was used that provided high energy density, superior rate performance, as well as long cycling life. Here, 1D nanofibers multichannel was designed through electrospinning to buffer the volume change, reduce the transport length of Lithium ions, and improve the matrix conductivity. These free-standing V_2_O_3_/MCCNFs deliver high capacity, excellent rate capability, and ultralong lifespan, along with the benefits like light weight, high-energy density, and remarkable cycling performance. The electrodes tested delivered superior capacity of 881.1 mAh g^−1^ at 0.1 A g^−1^ and 487.8 mAh g^−1^ at 5 A g^−1^ after 240 cycles and 5000 cycles with 0.00323% decay rate [167].

LIBs have pioneered battery technologies for decades with their high performance. However, there are still disadvantages such as toxicity and safety hazards due to their highly reactive properties and thermal runaway caused by dendrite formation, which has triggered research for mono and multivalent metal-ion battery systems. In past few years, magnesium-ion batteries (MIBs) have exhibited promising qualities due to the abundance in availability of magnesium and its ability to provide a volumetric capacity of 3832 mA h cm^−3^, which is twice as much of lithium. In their recent work, Diem and coworkers fabricated a hybrid magnesium–lithium-ion battery (MLIB) using a binder-free and self-supporting V_2_O_5_ nanofiber-based cathode with magnesium anode and dual-salt electrolyte containing both Mg^2+^ and Li^+^ [168]. V_2_O_5_ is a promising intercalation compound, as it can provide high storage capacities and energy densities owing to its redox chemistry. The presence of VO_5_ bilayer chains makes it a suitable cathode material for MLIBs. The space between the layers contain water molecules, which provides large vacancy to host intercalating ions and can facilitate ion insertion into intercalation sites. Here, V_2_O_5_ nanofiber cathode helped in achieving high energy densities by providing high intercalation potential. In this rechargeable electrochemical storage system, the cathode provided a high operating voltage of up to 1.5 V vs. Mg/Mg^2+^ while achieving storage capacities of around 386 mAh g^−1^, complemented with an energy density of 280 W h kg^−1^ and good cycling stability of 200 mA g^−1^ over 500 cycles.

Another hybrid battery study on a hygroelectric generator was carried out by Feng et al. [169] using an oxidized carbon nanofiber cathode and Mg anode based on the enhanced capacitive discharging effect. The concept of a water/moisture-induced hygroelectric effect depends on direct contact between magnesium alloy and oxidized carbon nanofibers in the presence of water (Figure 8b). Here, the CNF network acted as a good water-absorbing electrode. The reduction potential of Mg alloy was able to tune the oxidation of CNF and output voltage was made to exceed the limit while still generating a high current density.

The device could generate an open-circuit voltage up to 2.65 V and average peak short-circuit current density of ~6 mA/cm^2^ when in contact with liquid water, hence making it a potential candidate in water or vapor leakage detection, portable energy source, humidity sensors, etc. The fiber network in this study acted as a good water-absorbing material and could produce a high current density exceeding the reduction potential of magnesium by tuning the oxidation of the carbon nanofiber.

Other than Li and Mg batteries, Zn is another transition metal which has been highly explored in battery research for its high theoretical gravimetric energy (1086 Wh kg^−1^), easy conversion and storage, abundancy in nature, low cost, and environmental friendliness. Zang et al. [170] engineered NiO/NiCo_2_O_4_ porous nanofibers by electrospinning to use as bifunctional catalysts in the rechargeable Zinc air battery. NiO has been proven to be an ideal electrocatalyst in oxygen evolution reaction (OER)/oxygen reduction reaction (ORR) due to its special e_g_ orbitals, as the surface of nanoscale NiO is oxidized easily to active Ni^3+^ species. On the other hand, spinel structured NiCo_2_O_4_ has also been known as a promising OER, ORR electrocatalyst, as the cobalt and nickel ions can introduce various functions within the structure. In this engineered structure of NiO/NiCo_2_O_4_, chemical bonds that form at the interface facilitate a higher charge-transfer rate. In parallel, the heterostructure also promotes the intrinsic activity of OER/ORR through exposure of more electrocatalytic active sites. As a result, the interface engineering of NiO/NiCo_2_O_4_ with numerous mesopores synergistically provides sufficient oxygen transport, satisfactory intrinsic activity, and abundant electrocatalyst’ active sites. Here, NiO/NiCo_2_O_4_ porous nanofibers contributed to enhancing electron transfer rate and promoting the intrinsic activity through providing exposure to more electrocatalytic active sites. The synthesized catalyst exhibited superior electrocatalytic performance, lower overpotential of 357 mV at 10 mA cm^−2^, half-wave potential of 0.73 V, specific capacity of 814.4 mA h g^−1^, and cycling stability of 175 h.

Apart from the batteries, supercapacitors are another type of electrochemical energy storage devices that can store and release energy by reversible adsorption and desorption of ions at the electrode-electrolytes interface. High power density, rapid charge/discharge rate, and cycle stability are some of the key requisites of supercapacitors, which are supported by large surface area and high porosity of nanofiber material. The fibrous nanostructures surface morphology with high surface-to-volume ratio aids in increasing the energy storage capability by increasing interfacial activity between electrode and electrolyte [171,172]. A few of the latest research works in the field of nanogenerators are summarized here.

The development of supercapacitors with high energy density and rate capability is extensively explored for energy storage applications in electric vehicles and in wearable portable electronic devices. However, the conventional supercapacitors lack flexibility. To address this issue, Kim et al. [173] fabricated a metallized nickel platted carbon nanofiber (Ni@CNF) decorated with 2D Nickel Gallium sulfide (NiGa_2_S_4_) nanosheets. Here, NiGa_2_S_4_ nanosheets improved the electrochemical activity by promoting electrolyte-to-electrode diffusion. Along with this, superior electrical conductivity of the Ni@CNF provided electrochemical stability and also enhanced charge transfer that occurs in the electrode (Figure 8a). These nanofibers enhanced the pseudo capacitance and energy density of the nanosheets, whereas nanosheets contributed towards improving the energy storage capability and electrochemical activity. The device reported the highest specific capacitance at 488 F g^−1^ with a potential window of 1.1 V and current rate of 0.5 A g^−1^, long-term stability with capacitance retention 109% after 20,000 cycles, and flexibility effect on the cyclic voltammetry performance was tested by 2000 bending cycles, yielding favorable results. In another recent study, Jeong et al. [174] developed a template-less hierarchical porous carbon nanofiber/manganese oxide (MnO_2_)-derived electrospun polyacrylonitrile/cyclodextrin (CD) composite PMnCD(β) using β-CD phase, which exhibited a hierarchical porous structure. Hybrids of nano-sized MnO_2_ and carbon nanofibers have been declared as suitable candidates for supercapacitor electrodes, as they simultaneously exhibit high pseudo capacity and excellent electronic conductivity. The as fabricated PMnCD(β) electrode showed a large specific surface area for accumulation of ions and fast ion diffusion due to the mesopores and nitrogen groups. The β phase of CD nanofibers exhibited a hierarchical porous structure with a large specific surface area of 499 m^2^g^−1^, and 0.32 cm^3^g^−1^ total pore volume, that assisted in accumulation of hydrated molecules for double-layer formation by improving adsorption efficiency. Here. the electrode reported a maximum energy density of 25.3–16.0 Whkg^−1^ within the power density range of 400–10,000 Wkg^−1^, high specific capacitance of 228 Fg^−1^ at 1 mAcm^−2^, and excellent cycling stability of 94% after 10,000 cycles.

Nowadays, to further enhance the specific surface area, structural uniformity, porosity, functionality and diverse topology of supercapacitors, metal–organic frameworks (MOFs), and their derivatives are being integrated to the electrospun fibers network. Recently, Mukhiya and coworkers developed a 3D Co_3_O_4_/N-CNTs@CNFs for supercapacitors, exhibiting a promising performance through self-templated MOF-synthesized onto carbonized electrospun carbon nanofibers (CNF) mat. The composite supercapacitor exhibited a high specific capacity of 238 mA h g^−1^ at 1 A g^−1^ with a long lifespan and high rate capability. The assembled asymmetric supercapacitor could deliver 52.9 Whkg^−1^ specific energy at a specific power of 873.5 Wkg^−1^ outstanding lifetime (90.1% after 10,000 cycles) [175]. Apart from these, all-solid-state supercapacitors are becoming popular for their various advantages in terms of structural stability, operational safety, lightweight properties, and eco friendliness while also facilitating leakage prevention compared with the liquid electrolyte-based supercapacitors. By utilizing fiber-fiber interconnections, high specific surface area, and hierarchical pore structure of electrospun carbon nanofiber mats (ECNFMs), Wang and coworkers recently developed a high-performance all-solid-state supercapacitor. Here, ECNFMs were prepared by electrospinning of PAN and novolac resin (NOC) solution blend, where PAN was used as carbon precursor and NOC acted as a sacrificial agent contributing towards pore-generation. The highest obtained specific surface area value was 1468 m^2^g^−1^ when ECNFMs were carbonized at 1000 °C. A symmetrical supercapacitor device was prepared by sandwiching PVA/(Sulfuric acid) H_2_SO_4_ hydrogel within two freestanding ECNFMs. The device exhibited a high specific capacitance of ~99.72% along with excellent capacitance retention of ~320 mF cm^−2^ at 0.25 mA cm^−2^ after 10,000 charge/discharge cycles. Along with that, a large energy density of ~11.1 μWh cm^−2^ was obtained for input power density of 500 mW m^−2^ [176].

In another study, Singh et al. fabricated all-solid-state supercapacitors by kraft lignin-derived heat-treated carbon nanofibers. Although most of the studies use synthetic PAN polymer, its higher cost, non-renewable nature, and tendency to release toxic gases like HCN during heat treatment have promoted the usage of some renewable green precursors like Lignin, cellulose, etc. for synthesizing CNF. In this study, Lignin was selected for its abundancy in nature, non-toxicity, higher aromatic structure carbon content, and renewability. Higher specific capacitance 196.63 F/g was achieved for 1 A/g current density. For direct comparison, the device was tested in both aqueous (using 1 M H_2_SO_4_) as well as polymer gel electrolyte (using PVA-1M H_2_SO_4_ hydrogel). The solid-state electrolyte device could operate at higher voltages (exhibiting potential window of 0–2 V) compared with the aqueous electrolyte (where the potential window ranged within 0–1.6 V). Even after 10,000 cycles, the device exhibited 62.6 Wh/kg energy density and 1.25 kW/kg power density with 99.5% efficiency, providing futuristic energy storage applications of carbon nanofibers-based solid-state electrolyte [177]. Role of different nanofiber materials used in various type of batteries and supercapacitor applications are summarized in Table 5.

#### 3.2.2. Nanogenerators and Solar Cells

The nanogenerator is a portable energy-harvesting technology that converts small mechanical or thermal energy changes and produces electricity. Typically, nanogenerators are of three kinds based on the mechanism involved: piezoelectric (generating electric charge in response to applied mechanical deformation) [183], triboelectric, which is a combination of triboelectrification (the phenomenon of the certain class of materials to become electrically charged when brought in contact with their conjugate material with opposite tribopolarity) and electrostatic induction [184], and pyroelectric (generating electric charge in response to the changing thermal energy) [185]. Among these, triboelectric nanogenerators (TENGs) are being noticed for their high output voltage, simplified configuration, lightweight properties, cost-effective fabrication, flexible operation, etc. towards which nanofibers play a crucial role. Hence, they have been intensively explored in the past few years.

**Figure 9:**
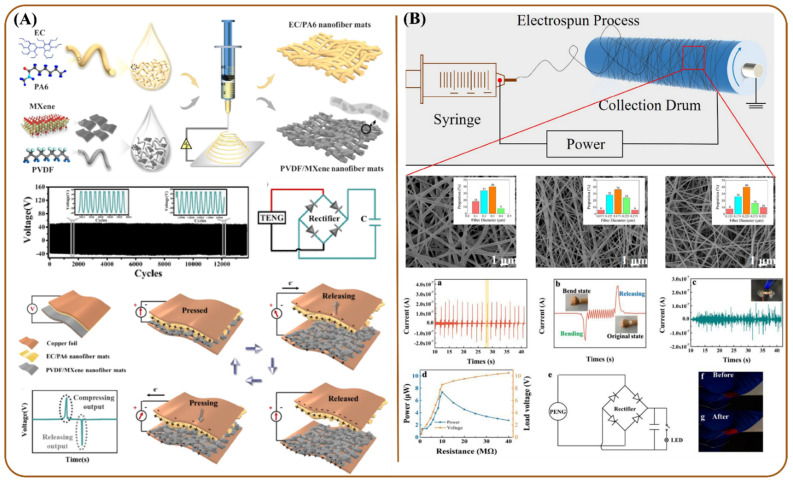
(**A**) Schematic of fabrication process, working mechanism, voltage, and current signals of TENG [186]. (**B**) Illustration of electrospinning system; SEM images and diameter distributions of PVDF fiber, (**a**) Short-circuit current of PENG with 2 wt% rGO under bending conditions. (**b**) One cycle short circuit current generated from the PENG with 2 wt% rGO. The inset shows the PENG with 2 wt% rGO mounted on the wrist with a bending and release gesture. (**c**) Short-circuit current of PENG with 2 wt% rGO under the condition of wind speed as 8 m/s. The illustration shows that the hair dryer continues to blow PENG. (**d**) Load resistance dependence of the output voltage and output power of the 2% rGO/PVDF PENG pressed at 2 Hz frequency. (**e**) Schematic illustration of rectifier circuit. (**f**) Before lighting the bulb. (**g**) The bulb was lighted [190].

To produce a scalable energy harvester for human motion monitoring and powering various light-emitting diode, Huang and coworkers used a simple one-step-electrospinning technique. Here, TENG was developed by electrospinning ethyl cellulose/polyamide 6 and poly(vinylidene fluoride) (PVDF) polymers (Figure 9A). In this study, ethyl cellulose (EC)/polyamide 6 (PA6) nanofiber served as a triboelectric positive material whereas PVDF incorporated with MXene sheet acted as a negative material. MXene has strong electronegativity and electrical conductivity, which boosts the triboelectric negativity of the PVDF nanofibers which, in turn, effectively improves the output of the TENG. EC has higher triboelectric positivity compared with PA6, but the tensile strength of EC nanofiber mats increases when it is blended with PA6, making EC/PA6 composite nanofiber mats a suitable candidate. The assembled TENG displayed stability, durability, and good output performance with peak power density of 290 mW/m^2^ at a 100 MΩ load resistance [186]. For composite nanofiber-based electrical devices, it has been reported that the fiber production technique also plays a crucial role in controlling their electrical as well as surface charge density. In their recent work, Kim and team developed polyimide/poly(vinylidene fluoride-co-trifluoroethylene) (PI/PVDF-TrFE) composite nanofiber membranes using single, conjugated, and multi-nozzle electrospinning systems [187]. Both PI and PVDF-TrFE have excellent triboelectric negativity as well as high dielectric constants. Here, combining PI as a transition layer with the PVDF-TrFE polymer contributed synergistically towards the TENGs performance. They reported that among the three types of systems, fibers obtained through the multi-nozzle technique exhibited the highest surface potential due to their thinner fiber diameter. During multi-nozzle electrospinning, PI nanofibers were placed adjacent to the PVDF-TrFE, forming well-developed crystalline structure resulting in a significant improvement in the electrical performances (obtaining stable energy harvesting up to 10,000 cycles of loading, with the ability to illuminate 117 light-emitting diodes) due to its high surface area. It was noted that the multi-nozzle setup delivered 364 V output voltage, 17.2 μA short-circuit current, and 29.72 nC transferred charge with power density of 2.56 W/m^2^ at a load resistance of 100 MΩ, which turned out to be about seven times greater than other nanofibers obtained from other setups.

Coaxial electrospinning is a technique mostly used for simultaneous electrospinning of solutions with poor dispersion and incompatibility. Zhang and group constructed a high-output-performance nanofiber TENG device comprising inorganic/organic hybrid materials to achieve synergic dielectric and dispersity modulation [188]. In this work, polydimethylsiloxane and barium titanate nanoparticles (BT NPs) were combined as the core and PVDF as shell layer of the coaxial nanofiber during the electrospinning process. BT NPs is a kind of inorganic piezoelectric ceramic that has excellent dielectric properties. It can effectively enhance the permittivity and surface charge density of corresponding triboelectric materials. PDMS is a widely used ideal tribo-negative material, and due to its good charge retention capability and presence of silicon hydroxyl end groups it possesses a better compatibility with BT than PVDF. The optimized device exhibits a high output voltage of 1020 V, a current of 29 μA, and a maximum power density of 2.2 W/m^2^ under a 30 MΩ load resistance. Due to their high energy conversion efficiency and ease of implementation, piezoelectric nanogenerators are extensively exploited as energy harvesting devices [189]. Designing piezoelectric devices with composite nanofiber is another popularly researched area in the nanogenerator field. Recently, Yang et al. [190] fabricated one of such piezoelectric devices using GO/PVDF electrospun nanofibers (Figure 9B). In PVDF polymer, β phase mainly contributes to the piezoelectric property of the device which becomes enhanced in the presence of external electrode polarization at high electric fields during electrospinning. Here, the addition of GO improves the content of β phase of PVDF by increasing nucleation sites. The device performance was further improved with the addition of reduced graphene oxide (rGO) which enhanced β-phase content, resulting in a 700 nA short-circuit current and 16 V open-circuit voltage. The higher conductivity of rGO facilitated improving the transfer of induced charge generated by PVDF, proving that rGO can enhance the piezoelectric response of the flexible fiber mat. Another recent work involved the use of PVDF/nickel ferrite (NiFe_2_O_4_) (400 nm diameter) nanofiber films [191]. Electroactive properties of polymeric nanocomposites were significantly influenced by the morphology of the nanofillers used. The larger aspect ratio formed a stronger interface with the matrix that improved the electroactive performance. Hence, the incorporation of NiFe_2_O_4_ within PVDF fiber resulted into transformation of microstructure from α-phase to β-phase by 68%. The fabricated device exhibited ferroelectric properties with a highest polarization value of 1.46 μC/cm^2^. In vibrational testing mode, the sample showed a maximum saturation magnetization value of 4.2 emu/cm^3^, open circuit voltage of 10 V, and 4.8 V output voltage under a weak AC magnetic field of 10 Oe at 50 Hz.

Apart from nanogenerators, solar (photovoltaic) cells are also energy harvesting device that converts light energy into electric energy. As the demand for cleaner energy is growing, solar cells, capable of producing renewable energy have been widely explored for their portability, efficiency, durability, and low maintenance. Nanofibers have been extensively used for solar cell research to achieve improved efficiency with other advantages like large scale production, miniaturization, etc. There are mainly three types of photovoltaic devices: dye sensitized, perovskite, and organic cells which have been explored recently [192]. A few of the recent research works are mentioned below.

Eco-friendly organic solar cells have several advantages, including biodegradability, safety, low-cost, lightweight properties, large surface areas, and nontoxic characteristics. Lin et al. successfully developed Ag NWs embedded TEMPO-oxidized eco-friendly cellulose nanofibers photovoltaic substrates using a facile, printable transfer method. In high-performance flexible OPVs, plastic flexible substrates like polyethylene terephthalate (PET) and polyethylene naphthalate (PEN) are mostly used, which have very high coefficients of thermal expansion that cause severe expansion or shrinkage, resulting in cracks. To avoid such damages, TEMPO-oxidized Cellulose NFs were used here. It was observed that the TEMPO-mediated oxidation largely improved mechanical strength of CNFs. Here, the nanofiber network enabled the formation of a homogeneous hybrid with Ag NWs and concurrently allowed a higher percentage of visible light. The device showed a low sheet resistance of 2.62 Ω sq^−1^ with 78.5% optical transparency of (at 550 nm), which was comparable to the commercial polyethylene naphthalate/indium-tin-oxide substrate. The device was able to withstand 15 peeling cycles and 500 bending cycles, displaying its high mechanical stability and very low thermal expansion coefficient (3.37 × 10^−6^ K^−1^) that aided in obtaining a new perspective for the development of sustainable organic photovoltaics [193].

In spite of many advantages, there are few drawbacks such as low efficiency, stability, and strength, which triggered research interest in looking for other alternatives. A recent study on iron platinum (FePt)/Titanium dioxide (TiO_2_) nanofibers-based solar cell was conducted by Nien et al. [194]. The device was fabricated onto the photoanode material for dye-sensitized solar cell using sol-gel and electrospinning technique. Here, TiO_2_ nanofibers acted as a barrier layer which reduced the charge transfer resistance by preventing recombination of photogenerated electrons and holes. As FePt nanoparticles exhibit high chemical stability and coercivity in addition to their excellent magnetic property, they can reduce the surface charge recombination rate of metal oxides. Hence, in this study, FePt nanoparticles were mixed with TiO_2_ nanofibers to modify the photoanodes. The device showed an output intensity of 100 mW/cm^2^ and a 16% increase in photoelectric conversion efficiency when compared with the TiO_2_ photoanode, which was 3.79%, and FePt/TiO_2_ nanofiber photoanode, which was 4.41%. In general, solar cell transparency is a crucial factor to capture and utilize the solar energy. In this direction, a study involving fabric-like transparent electrode for flexible perovskite solar cell was carried out by Zhai et al. [195]. In this work, a silver nanowire network-loaded PU/PAN nanofibers film was fabricated. Here, a nanofiber film of approximate thickness of 10 µm provided high transmittance and served as an excellent flexible electrode. It was noticed that with increasing PAN content, although the breaking strength enhanced, the light transmittance value decreased. The produced electrode possessed 80% light transmission in the visible range, and 70% break strain while retaining its original charge transport property during stretching and bending operations. Hence, employing this electrode to assemble perovskite solar cells resulted a 4.06% conversion efficiency while maintaining above 85% of its original efficiency.

The roles of different nanofiber materials used in various nanogenerator applications are summarized in Table 6.

## 4. Conclusions

This article was structured and presented in a format to provide an understanding of the state-of-the-art properties, characteristics, and advanced applications of nanofiber technology. Nanofibers with high surface area and tunable porosity, along with other novel properties like high conductivity, superior electrochemical activity, improved mechanical strength, structural integrity, etc., are being fabricated through the unique, simple, lucrative, and versatile technique of electrospinning. The ability to affect nanofiber morphology with respect to the electrospinning setup modifications, along with environmental, solution, and process parameters, was discussed in the review. Applications including biosensors, tissue engineering, wound healing, and drug delivery in health care sector and batteries, supercapacitors, solar cells, and nanogenerators in energy devices were elaborated in this article. Although electrospinning is the most versatile among existing techniques, there are a few drawbacks such as: requirement of high voltage and conducting targets; difficulty in achieving in situ deposition; scalability for mass production; requirement of specialized equipment and reproducible systems for commercialization; difficulty in obtaining long, continuous, and oriented nanofiber; avoiding beads formation during electrospinning caused by solution viscosity or net charge density that reduces effective surface area of fibers; becoming friable after calcination; and difficulty in fabricating nanofibers with diameters less than 10 nm, etc. Along with these, there are also application-specific shortcomings for each field. In health care applications, despite having unique properties, electrospun nanofibers show a low level of biodegradability, poor infiltration of cells or drugs into the scaffolds, and a consistency mismatch between the nanofibers and bone extracellular matrix, etc. Similarly, some challenges have also been faced in the applications of electrospun nanofibers-based energy devices. Some of these are inefficient inhibition, unavailability of efficient and durable redox activation and stability, limited theoretical specific capacity, the need of improved energy density, reproducibility, extended shelf life, and longer cycle life, etc.

The drawbacks and challenges that are being faced currently have become the problem statement for future research works. Commercialization and meeting the demand of nanofiber technology have become prime focuses for future research which aim towards large-scale production for facilitating laboratory-to-market transition. The brittleness of electrospun nanofibers is also a major setback for various applications, which has become another triggering factor for upcoming research, especially on developing highly flexible continuous fibers. To obtain synergistic chemical as well as physical characteristics, the addition of nanofillers within the fiber matrix has become another key researched area for both biomedical and energy related applications. In the case of tissue engineering, wound dressing, and drug delivery, qualitative to quantitative analysis for cytocompatibility of the fibrous scaffolds for cell adhesion, proliferation, and differentiation is becoming an essential feature. Along with this, exploration through in vivo animal/human study with clinical trials for evaluating real-life practical applicability of the device or scaffold should also be emphasized. On the other hand, in energy devices, synthesizing nanofiber-based electrodes with optimized porosity and pore distance for improving ion carrying and storing capacity is one of the upcoming major goals which will subsequently enhance both voltammetric energy and power density. The aim of this review was to provide an overall insight on electrospun nanofibers and their advanced applications through understanding the direction of research that has been conducted in the past 2 years in the fields of health care and energy devices.

## Figures and Tables

**Table 1:** Role of nanofiber materials in various biosensing applications.

Polymer Type	Materials	Property and Role	Applications	Ref.
Natural polymer	Cellulose Monoacetate/Tetraethyl Orthosilicate Hybrid Nanofibers	Monitored guanine base oxidation in single-strand DNA by electrochemical methods	Electrochemical DNA biosensor	[103]
Natural polymer	Cellulose acetate nanofiber	CA/ZIF-8@enzyme/MWCNTs/Au membranes were utilized as highly flexible electrodes	Glucose biosensor	[105]
Synthetic polymer	Activated multiscale pore contained carbon nanofiber (a-MPCNF)	Signal transducer and template for immobilization of nesfatin-1 antibody	FET (field-effect transistor)-based biosensor	[102]
Synthetic polymer	In-Ga-Zn-O (IGZO) nanofiber	Facilitated increase in the sensitivity without adjusting the gate-oxide materials or thickness	FET-based chemical and biosensor	[111]
Synthetic polymer	Polyacrylonitrile/ Polypyrrole Core-Shell Nanofibers	Facilitated covalent binding of the enzyme, glucose oxidase	Glucose biosensor	[106]
Synthetic polymer	Copper nanoflower decorated Au NPs graphene oxide nanofiber	Enabled conversion from glucose to gluconic acid through electro-catalyzed reaction while improving the electrochemical properties	Glucose biosensor	[107]
Synthetic polymer	ZnO-CuO nanofibers	Tuned nanostructure for improved amperometric detection of H_2_O_2_.	Non-enzymatic biosensor	[110]
Synthetic polymer	SnO_2_ nanofiber	β-Amyloid (1–42) is a viable biomarker for early diagnosis of Alzheimer’s Disease. Specific capture antibody was covalently immobilized on the SNF nanofiber-modified carbon working electrode for immunoassay	Immunosensor	[108]
Synthetic polymer	Co_3_O_4_ doped carbon nanofiber	Combined the high electrochemical conductivity and good biocompatibility with novel synergistic effects for the immobilization of redox protein.	Hemoglobin-based electrochemical sensor	[112]
Synthetic polymer	Lignin/PAN carbon nanofiber	Carbon nanofiber and graphene reinforced carbon nanofiber could increase the conductivity of the screen-printed electrode. Biosensor for specific recognition of biomolecules with graphene reinforced carbon nanofiber modified screen-printed electrode was fabricated for the first time.	Acetaminophen Biosensor	[113]

**Table 2:** Role of nanofiber materials in various tissue engineering applications.

Materials	Property and Role	Applications	Ref.
Platelet-rich fibrin loaded/chitosan core-shell nanofibrous scaffold	Helps in protecting and preserving the biomolecules from direct contact with solvents and changing microenvironment during in vitro studies and in vivo implantation.	Bone tissue engineering	[124]
Polycaprolactone/polyvinylpyrrolidone nanofibers	Attachment and proliferation of osteoblasts in vitro while potentially enhancing the cell–cell interactions and cell migration necessary for bone healing	Bone regeneration	[121]
Polylactic acid glycolic acid/silk fibroin membranes loaded with artemisinin	Polylactic acid glycolic acid can provide good mechanical properties, and SF can improve the biocompatibility and drug-release property of dressings. ART was loaded on membranes as an anti-inflammatory agent	Wound dressing	[130]
Poly-ε-caprolactone (PCL)/whitlockite (WH) nanofiber membrane	This biomimetic nanofiber membrane combines the positive osteogenic differentiation ability and angiogenic ability of calcium phosphate materials, and is expected to be used for artificial periosteum.	Periosteal Regeneration	[123]
Poly(oligoethylene glycol methacrylate)/cellulose nanocrystal hydrogel nanofibers	Nanofiber alignment played a supporting role on cell alignment, with parallel-oriented fibers further promoting cell orientation with respect to the underlying uniaxial wrinkle direction while perpendicular-oriented fibers locally de-aligned the cells.	In vitro cell screening and in vivo tissue regeneration	[131]
Gelatin methacryloyl-coated, 3D expanded nanofiber scaffolds	These scaffolds seeded with various types of cells, including dermal fibroblasts, bone marrow-derived mesenchymal stem cells, and human neural stem/precursor cells to form 3D complex tissue constructs.	Tissue regeneration	[132]
Poly(l-lactide-co-ε-caprolactone)/silk fibroin scaffolds	Supports in mimicking the microarchitecture of native cartilage.	Cartilage regeneration	[122]
Polyamide-6/chitosan nanofibrous membranes	The nanofibrous membrane provides high toughness and good mechanical properties (tensile strength 1.41 ± 0.18 MPa and elastic modulus 7.15 ± 1.09 MPa) and supports bioactivity, biocompatibility and osteoconductivity.	Bone regeneration	[133]
Poly(4-hydroxybutyrate) nanofiber membrane	The electrospun P4HB membrane could facilitate fibroblasts infiltration in the porous structure, with a proper degradation rate that matches the new tissue formation during dural re-construction.	Tissue regeneration	[126]
Poly (l/dl-lactic acid nanofiber)	When functionalized with a chemotactic agent such as the SDF-1α/CXCL12 chemokine, an in-situ increment of migration signaling on the surface to drive cells through the fibers was achieved	Neural Regeneration	[129]

**Table 3:** Role of nanofiber materials in various drug delivery applications.

Materials	Property and Role	Applications	Ref.
Small intestine submucosa/poly(ε-caprolactone-ran-L-lactide) Nanofiber Sheets	Facilitated controlled drug delivery related to anti-inflammation	Drug delivery	[135]
Poly (vinyl alcohol)/soy protein isolate (PVA/SPI) nanofiber mats	The mats were loaded with ketoprofen by dissolving the drug in the solutions for nanofiber electrospinning, providing channelization.	Drug delivery	[136]
Pluronic F-127-polycaprolactone nanofiber mats	Nanofibrous core helped in encapsulating antimicrobial agents with a hydrophilic nature	Drug delivery	[137]
Ibuprofen (IBU)-loaded polyvinylpyrrolidone nanofibers	Concurred fine functional performances of electrospun nanofibers on improving the dissolution of IBU	Drug delivery	[139]
Thiram/hydroxypropyl-β-cyclodextrin inclusion complex electrospun nanofibers	Improved the water solubility of thiram	Drug delivery	[143]
5-fluorouracil (5-FU) loaded Eudragit S-100 composite nanofibers	The developed nanofiber formulation exhibited superior tumor regression potential and pharmacokinetic profile compared with the plain drug	Drug delivery	[142]
Core-shell nanofibrous system containing vancomycin and imipenem/cilastatin	The nanofibers showed significant activity against both gram-positive and negative bacteria, causing diabetic foot ulcers.	Drug delivery	[138]
Poly(vinyl alcohol) and chitosan incorporated with moxifloxacin hydrochloride nanofibers	Nanofibers exhibited good antibacterial properties against *Staphylococcus aureus* and *Pseudomonas aeruginosa* due to the moxifloxacin hydrochloride incorporation.	Drug delivery	[144]
Sulindac vinyl alcohol-co-ethylene nanofiber membrane	Nanofiber membranes demonstrated characteristics of high drug loading and stability	Drug delivery	[145]
CuS composite nanofibers	These CuS composite nanofibers could be deposited in situ on the wound to simultaneously achieve rapid hemostasis and ablate superbacteria of PA without using extra materials and devices	Drug delivery	[140]

**Table 4:** Role of nanofiber materials in various wound-dressing applications.

Materials	Property and Role	Applications	Ref.
*Blumea balsamifera* oil loaded cellulose acetate nanofiber mats	The oil loaded nanofiber mats showed good antibacterial efficacy against the *E. coli* and *S. aureus* in agar disc diffusion and OD (bactericidal effect with time-dependence) tests	Wound dressing	[151]
ciprofloxacin-loaded chitosan/polyethylene oxide/silica nanofibers	For the first time, this hybrid organic/inorganic material was introduced for wound dressing and the in vitro and in vivo preclinical examination of the prepared nanofiber.	Wound dressing	[152]
Cellulose acetate/annatto extract nanofibers (CA/annatto)	Cellulose acetate nanofibers containing crude annatto extract potential for biomedical applications	Wound dressing	[153]
Lidocaine hydrochloride (LID) and mupirocin-loaded chitosan/polycaprolactone (CSLD-PCLM) Nanofiber scaffolds	The nanofiber structure enhanced the interfacial interaction between the scaffold and blood cells	Wound dressing	[155]
Cerium-doped bioactive glass-loaded chitosan/polyethylene oxide nanofiber	Scaffold in its wet state had mechanical properties very close to the skin and its elongation at break was 28.6%, which was only 20% less than the required elongation at break for skin tissue scaffolds.	Wound dressing	[158]
Curcumin loaded polycaprolactone-/polyvinyl alcohol-silk fibroin nanofibers mat	Polycaprolactone and polyvinyl alcohol helped to strengthen the nanofiber	Wound dressing	[159]
Chitosan -polycaprolactone/polyvinylalcohol (PVA)-melatonin (MEL)/chitosan-polycaprolactone three-layer nanofiber	The three-layer wound dressing reduced burst release (45%) and led to a sustained release of melatonin.	Wound dressing	[157]
Starch/AgNPs composite nanofiber mats	Starch/AgNPs composite nanofiber mats altered cytotoxicity and antibacterial activity	Wound dressing	[160]
Chitosan/poly (vinyl alcohol) nanofiber membrane	Nanofiber membrane crosslinked with the blocked diisocyanate to enhance water resistance and mechanical properties of nanofiber membrane. Antibacterial efficacy of nanofiber membranes reached ~100%	Wound dressing	[161]
Polycaprolactone/chitosan oligosaccharide nanofiber	The blended chitosan oligosaccharide in PCL increased membrane hydrophilicity.	Wound dressing	[162]

**Table 5:** Role of nanofiber materials in various battery and super capacitor applications.

Materials	Property and Role	Application	Ref.
ANF/PEO-LiTFSI CPE films	Provided large aspect ratio and high mechanical strength, and conductivity. The CPEs also displayed greatly enhanced electrochemical, mechanical and thermal stabilities with superior rate performance and cycling stability	Li-ion battery	[166]
Vanadium oxide/multichannel carbon nanofibers	Exhibited unprecedented electrochemical rate performance, long cyclability	Li-ion battery	[167]
Zeolitic imidazolate framework-67/cellulose nanofibers	Displayed improved pore structure, excellent thermal stability, lower thermal shrinkage, and better surface wettability. Lithium-ion batteries assembled with ZIF-67@CNF membrane showed higher cycling performance and rate capability than the other membranes	Li-ion battery	[178]
Binder-free and self-supporting V_2_O_5_ nanofiber	Cathode materials: high storage capacities, good cycling stability while holding structural integrity	Magnesium–lithium-ion hybrid battery	[168]
Carbon nanofibers (CNF)	The oxidized CNF was shown to absorb water/moisture and became reduced, leading to a capacitive discharging effect to provide enhanced signal amplitude and sensitivity	Magnesium battery	[169]
Porous NiO/NiCo_2_O_4_ nanofibers with	Provided flexibility and long cycling life (14 h)	Zinc-air battery	[170]
Nitrogen-doped porous carbon @Carbon nanofiber aerogels	Exhibited peak density of 96 mW cm^−2^ and stable charge discharge durability	Zinc-air battery	[179]
Vanadate nanofiber crystal structure/poly(3,4-ethylene dioxythiophene)	Intercalation of the conducting polymer increased electron pathway, in turn increasing number of active sites inside the vanadate and accelerating the zinc ion intercalation/de-intercalation process.	Zinc ion battery	[180]
Lithium iron phosphate/Carbon nanofibers composite	Exhibited higher specific discharge capacity, high rate performance, and better cycling values	Li-ion battery	[181]
3D flexible freestanding nitrogen-doped carbon nanofiber/MoS_2_ nanoflowers (NCNFs/MoS2) network	Provided a highly conductive pathway for the fast transfer of charge and electron, and expanded interlayer spacing of MoS_2_ nanoflowers while increasing available surface-active sites by reducing the obstacle for Li-ion Li-ion migration during lithiation/delithiation process	Li-ion battery	[182]

**Table 6:** Roles of nanofiber materials in various nanogenerator applications.

Materials	Property and Role	Applications	Ref.
Ethyl cellulose/polyamide 6 nanofiber mats and MXene-based poly(vinylidene fluoride) nanofiber mats	Ethyl cellulose/polyamide 6 nanofiber mats as triboelectric cathode materials and MXene-based PVDF nanofiber mats as triboelectric anode materials.	Triboelectric nanogenerator	[186]
Polyvinylidene fluoride (PVDF)/MXene (Ti_3_C_2_T_x_) composite nanofibers	The dielectric modulation of PVDF nanofibers by incorporating conductive MXene nanosheets significantly enhanced the dielectric constant and the surface charge density of nanofiber by 270% and 80%, respectively.	Triboelectric nanogenerator	[196]
Polyimide/poly(vinylidene fluoride-co-trifluoroethylene) membranes	Provided excellent triboelectric negativity and high dielectric constant	Triboelectric nanogenerator	[187]
Polyamide 66 nanofiber and poly(vinylidenefluorideco-trifluoroethylene) nanofiber	Nanofibers were used as the shell to wrap commercial stainless-steel yarns providing high flexibility, desirable breathability, washability, excellent durability, demonstrating to be a reliable power textile to light up 58 light-emitting diodes	Triboelectric nanogenerator	[197]
Graphene Oxide/PVDF Electrospun Nanofiber	By adding GO and rGO to increase the nucleation sites in PVDF, the polar β piezoelectric phase could be enhanced, resulting in greater piezoelectric output	Piezoelectric nanogenerator	[190]
PVDF-graphene nanosheet composite nanofibers	In comparison with the pristine PVDF, the PVDF/G composite films-based TENGs demonstrated superior triboelectric performance.	Triboelectric nanogenerator	[198]
PVDF/ZnO composite nanofibers	Electrospraying pre-synthesized ZnO nanorods on PVDF nanofibers resulted in the highest piezoelectric response due to the combined effect of the greater piezoelectricity of aligned ZnO nanorods and PVDF nanofibers, and larger triboelectric response from increased surface roughness.	Piezoelectric nanogenerator	[199]
Poly(vinylidene fluoride-trifluoroethylene) (PVDF-TrFE)/MXene Nanofiber Mat	The incorporation of MXene nanosheets into the PVDF-TrFE polymer matrix substantially increased electronegativity in the composite polymer that helped the dramatic enhancement in the performance of the nanofiber-based triboelectric nanogenerator	Triboelectric nanogenerator	[200]
Poly(vinylidene fluoride)(PVDF)/nickel ferrite (NiFe_2_O_4_) fiber	Addition of NiFe_2_O_4_ in PVDF plays an important role in transforming α to β phase. PVDF/NiFe_2_O_4_ films exhibited enhanced ferroelectric and magnetic properties.	Piezoelectric nanogenerator	[191]
Polydimethylsiloxane (PDMS)/ barium titanate@ poly(vinylidene fluoride) (PDMS/BT@PVDF) core-shell nanofibers	PVDF as the outer layer and PDMS/BT as the core where liquid PDMS was crosslinked in situ by employing a heating plate collector.	Triboelectric nanogenerator	[188]

## Data Availability

Not applicable.
